# No evidence for disruption of empathy and mentalizing by face coverage in multimodal settings

**DOI:** 10.1038/s41598-025-25393-7

**Published:** 2025-12-05

**Authors:** Eva Landmann, Inga Felicia Straub, Anne Böckler

**Affiliations:** Department of Psychology, Julius-Maximilians-Universität Würzburg (JMU), 97070 Würzburg, Germany

**Keywords:** Human behaviour, Psychology

## Abstract

In social interactions, we often encounter situations where a partner’s face is (partially) occluded, e.g., when wearing a mask. While emotion recognition in static faces is known to be less accurate under such conditions, we investigated whether these detrimental effects extend to empathic responding, mentalizing (i.e., Theory of Mind), and prosociality in more naturalistic settings. In four studies (N_total_ = 157), we presented short video clips of narrators recounting neutral and emotionally negative autobiographical stories, with their faces shown in four conditions (two per experiment): fully visible, eyes covered, mouth covered, and audio-only. Participants then responded to questions assessing affect, mentalizing performance, and willingness to help. Affect ratings were slightly lower when the narrator’s mouth was covered, and participants were less willing to help narrators with covered eyes. Importantly, however, empathic responding and mentalizing performance remained robust across visibility conditions. Thus, our findings suggest that social understanding – specifically, empathizing and mentalizing – is not substantially impeded by partial or complete facial occlusion, when other cues, such as vocal information, can be used to compensate. These insights may help contextualize concerns about detrimental effects of face coverage in social interactions.

**Supplementary Information:**

The online version contains supplementary material available at 10.1038/s41598-025-25393-7.

## Introduction

As long as humans have been interacting, they have encountered circumstances that prevent them from fully perceiving those they are engaging with. The ancient myth of Pyramus and Thisbe describes the star-crossed couple whispering love confessions through a crack in the wall to evade their feuding families^1^. We, too, as citizens of the less romantic 21st century sometimes find ourselves engaging with others whose face is partially covered, whether by face masks or sunglasses. Does this impair our ability to empathize with them and to correctly understand their beliefs and intentions? After all, Pyramus and Thisbe met a tragic end that was (in some way) caused by a misunderstanding. And during the Covid-19 pandemic many worried about a decline in social understanding due to the widespread use of protective masks^2,3^. With only part of the facial information available, people might fail to recognize the feelings and needs of the ones surrounding them, ultimately fostering a lack of connection and maybe even prosocial behavior. Addressing this concern, we sought to investigate whether partially or fully occluding a target person’s face does in fact have a detrimental impact on empathy, mentalizing, and prosociality.

Numerous studies have examined how the reduction or absence of face cues impacts emotion recognition. While pre-pandemic experiments utilized pieces of cardboard^4^, different types of headdress^5,6^, sunglasses^7^, as well as bars and other geometric shapes^7^ to reduce the visibility of facial features, many studies from 2020 onward specifically focused on face masks^8–17^. Throughout these studies, a consistent pattern emerged: When parts of the face were covered, emotional expressions were recognized with significantly lower accuracy, affecting both the categorization of the emotions and the assessment of their intensity. Visibility of the mouth area, in particular, was identified as crucial for accurate recognition^18,19^, although the informative value of different face areas varies depending on the specific emotion^20–23^. In addition to impeding emotion recognition, occluding parts of the face seems to impair face identification^10,15,24^ and reduce assessments of interpersonal closeness^11,25^. Taken together, these findings indicate that a lack of available facial cues may impair social understanding, aligning with concerns regarding face mask usage.

However, almost all of the referenced studies employed static images or very short video stimuli with little or no context (for an exception see^25^). While there may be some real-world scenarios where these conditions apply, such as briefly glimpsing the masked face of a person driving by, they do not match the complexity of most meaningful real-life interactions^26,27^. Facial expressions, although prominent^23,28–30^, are not the only manifestations of emotions, and affective states are also conveyed through vocal^31–33^, bodily^34–36^, and olfactory^37–39^ cues. Given this plethora of abundant (and redundant^40^) signals, it is not far-fetched to assume that humans are able to compensate for a lack of visual information in realistic interaction settings. On the contrary, it is hard to imagine that one would not understand and feel for a heartbroken friend burying their head in a pillow, while still seeing their trembling shoulders and hearing the anguish in their voice as they talk about the barriers separating them from their soulmate.

Besides anecdotal experience, there is considerable empirical support for humans’ capability to flexibly infer another person’s mental state from signals other than facial information. Motion cues, for example, appear to hold the potential to compensate for incomplete or compromised information in emotion recognition (for a review see^30^). With regard to face masks specifically, the aforementioned negative effects on emotion recognition were largely mitigated when the person’s full body was visible^41^. Moreover, studies using longer video clips of masked and unmasked target persons talking about themselves and their (emotional) experiences found no reduction in social connectedness and empathic accuracy^42,43^. This suggests that in dynamic, contextualized, and multimodal settings – more closely mirroring real-life interactions – the detrimental effects of face masks are much less severe or even absent. In these scenarios, the voice of one’s interaction partner might provide valuable insights into their inner workings both through the content of their words and the manner of speaking. While combining face and voice information typically leads to the best emotion recognition performance^44–46^, accuracy rates are still high when using voice-only cues^31,47,48^ and audio stimuli can effectively evoke empathic responses and accurate mentalizing^49^. In studies presenting videotaped conversations, verbal information was identified as the most essential source for accurate understanding, especially when inferring a person’s thoughts (rather than their feelings)^50,51^. Some researchers even suggested that voice-only communication enhances empathic accuracy by reducing cognitive load and enabling a more effective focus on highly informative cues provided by the voice^52^.

The opposing findings and conclusions may seem difficult to reconcile at first, but they can be at least partially understood by considering the differences in methodology. We have already pointed out the use of static, isolated cues compared to longer and contextually enriched stimuli, which offer less experimental control but higher ecological validity. Social cognitive processes like the perception of (emotional) faces are known to vary between naturalistic and more controlled experimental settings^53,54^, with real-life approaches frequently suggesting conclusions that differ from those originally assumed based on less ecologically valid paradigms^55^. In addition, the referenced studies addressed social understanding by means of a wide range of dependent variables, from basic emotion identification to complex measures of empathy and mentalizing, also referred to as Theory of Mind (ToM). This distinction is critical, as reduced face visibility may impede the quick recognition of prototypical emotion expressions, but not the tendencies to empathize, mentalize, and behave appropriately – arguably the most crucial aspects in real-life interactions. In our study, we employed dynamic video clips resembling realistic social interactions, implementing varying levels of face occlusion, and focused on two central functions of social understanding that go beyond emotion recognition: *empathizing*, defined in our context as the sharing of another person’s emotions^56,57^, and *mentalizing* (or, used interchangeably, ToM), which encompasses cognitive reasoning about a person’s thoughts, beliefs, and intentions^58,59^. The inclusion of affective as well as cognitive components enables us to draw more nuanced conclusions about which facets of social understanding (if any) are affected by a lack of facial information. Furthermore, we consider these two functions highly relevant due to their influence on *prosociality*, as both empathy and ToM have been linked to costly helping, donations, and other forms of cooperative and prosocial behavior^60–64^.

**Fig. 1:**
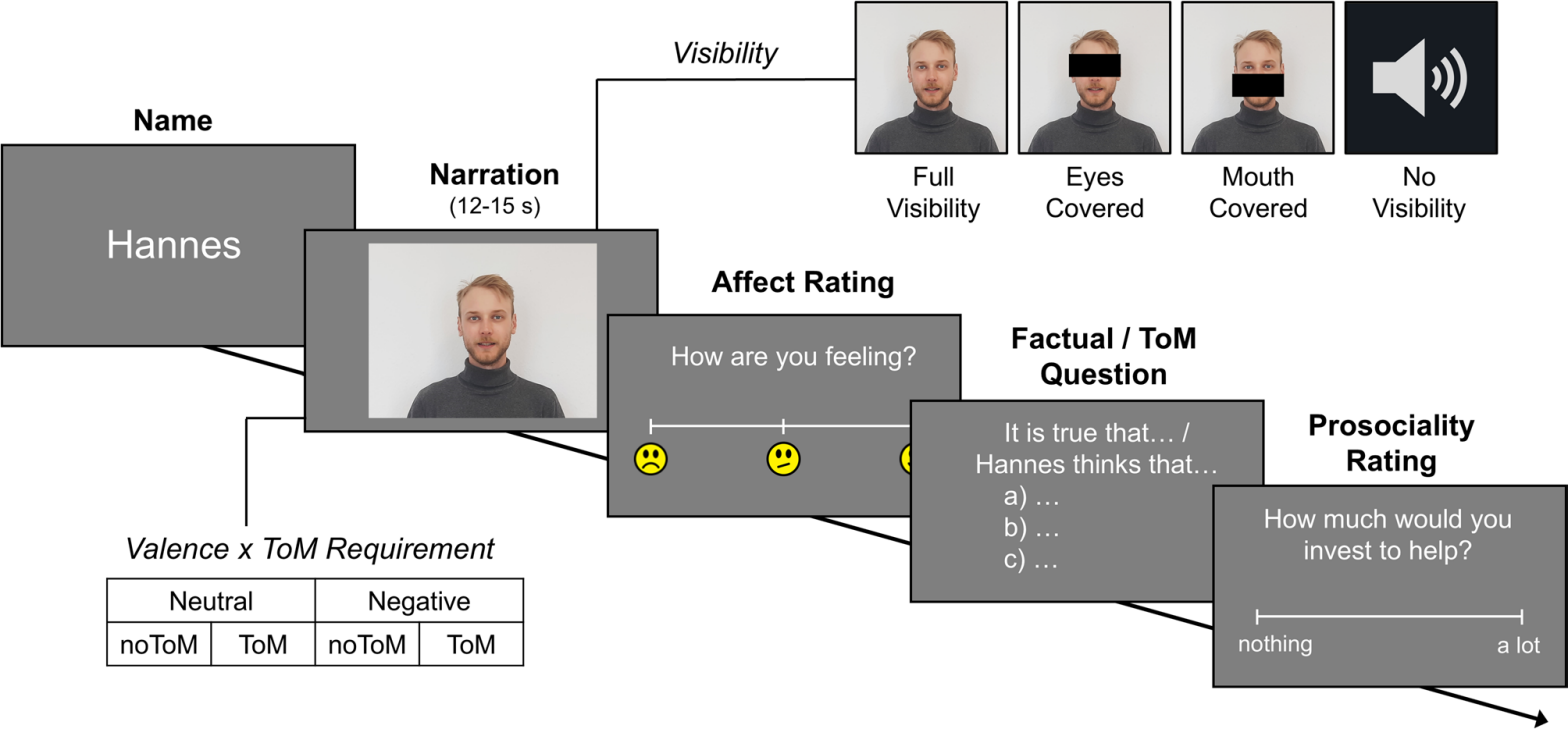
Schematic depiction of the experimental procedure and conditions. The narrations were either emotionally neutral or negative (Valence) and either required ToM or factual reasoning to answer the question they prompted (ToM Requirement). In each experiment, clips were presented in two of four Visibility conditions. Exp.1: full visibility vs. eyes covered, Exp.2: full visibility vs. mouth covered, Exp.3: eyes covered vs. mouth covered, Exp.4: full visibility vs. no visibility (audio-only). Each participant completed 48 trials in a fully crossed 2 × 2 × 2 within-subjects design. The individual in the recreated video stills provided informed consent for publication of the images in an online open-access publication.

The objective of our study was to investigate if and how partial or complete occlusion of an interaction partner’s face affects empathizing, mentalizing, and prosociality, in a task using naturalistic, dynamic, and multimodal stimuli. Following the established EmpaToM paradigm^65^, participants watched video clips of narrators recounting brief autobiographical stories (for a schematic depiction of the experimental procedure see Fig. 1; for example narrations see Supplement S1) about either neutral or negative experiences (*Valence*: neutral vs. negative), that either did or did not require ToM (*ToM Requirement*: noToM vs. ToM). After each clip, we assessed: (i) participants’ current affective state (*affect rating*), interpreting the difference in ratings between neutral and negative narrations as empathic responding; (ii) their accuracy (*question accuracy*) and – as a complementary measure – response time (*question RT*) in answering a question about the narration, which either required factual reasoning (in noToM trials) or mentalizing (in ToM trials); and (iii) their willingness to invest (hypothetical) resources to help the person in the video (*prosociality rating*).

Critically, we manipulated *Visibility* of the narrator’s face across four experiments with a total sample size of *N* = 157 (for an overview of the experiments, see Table 1). Each experiment compared two of a total of four conditions: In the *full visibility* condition, participants saw the narrator’s face without any restrictions, replicating the original EmpaToM. In the *mouth covered* condition, we added a black bar to obstruct the visibility of the mouth area. In the *eyes covered* condition, we did the same for the eye area. Finally, in the *no visibility* condition, participants saw a blank screen and could only hear the narration (audio-only). We opted for the somewhat artificial coverage using black bars over more naturalistic obstructions like masks and sunglasses in order to maintain high comparability across conditions and to prevent the type of coverage and associated attitudes from influencing the results^8,66^. Therefore, any effects emerging in our data can be solely attributed to the visibility manipulation.

Based on the mixed state of research, we considered several plausible result patterns (for more detailed hypothesis derivations, see also the following preregistration, which was created as part of a student project for two of the experiments: 10.17605/OSF.IO/DS4VE): If the reduction of available facial information significantly impairs the components of social understanding, as evidenced for emotion recognition^4,9,11,67^, we would expect diminished empathic responses – reflected in smaller affect rating differences between neutral and negative narrations – as well as reduced mentalizing performance (especially response accuracy), and prosociality under conditions of limited facial visibility. The extent of this reduction might be contingent on the degree of coverage – the less facial information available, the higher the impact – or on the specific face areas covered. One possibility is that occluding the mouth, frequently considered the most diagnostically informative feature of the face^18,19^, could particularly present difficulties. Alternatively, one could hypothesize that within our study, it is more detrimental to cover the eye area, given that the predominant emotion in the negatively valenced narrations is sadness – an emotion for which the eyes are assumed to provide the most informative cues^21,23,68^. However, an entirely different prediction presents itself if one takes into account that humans are highly proficient in deducing another person’s thoughts and feelings from a variety of signals – vocal cues in particular^32,40,51^ – and can compensate for the missing visual input in multimodal settings. With this in mind, we would not expect differences in empathic responding, mentalizing accuracy, and prosociality as a function of face visibility. Lastly and given the established differentiation between empathy and ToM^69,70^, it is plausible that the results could vary between these functions. For instance, face occlusion might more severely impact empathic responses, where emotion recognition is crucial^71–73^. Determining which of these hypothesized patterns emerges in our data will offer more comprehensive and nuanced insights into the potential impairment of social understanding when facial cues are absent or only partially available.

**Table 1:** Overview of the experiments and demographic data of the participants. Full *N*: Number of participants who completed the experiment. Final *N*: Number of participants included in the main analyses (excluding those not meeting the accuracy criterion).

	Visibility conditions	Full *N*	Final *N*	Mean age (SD)	*n* female
Exp. 1	Full Visibility/Eyes Covered	41	38	25.2 (8.3)	31
Exp. 2	Full Visibility/Mouth Covered	41	40	21.6 (3.9)^a^	32^a^
Exp. 3	Eyes Covered/Mouth Covered	43	41	27.1 (8.7)^b^	29^b^
Exp. 4	Full Visibility/No Visibility	40	38	25.6 (5.3)	30
Total		165	157		

^a^ Demographic data missing for two participants.

^b^ Includes demographic data of one participant retroactively excluded from the main analyses.

## Results

For each of the four experiments, we computed linear mixed models (LMMs) with fixed effects for Valence (neutral vs. negative), ToM Requirement (noToM vs. ToM), and Visibility (Exp.1: full visibility vs. eyes covered; Exp.2: full visibility vs. mouth covered; Exp.3: eyes covered vs. mouth covered; Exp.4: full visibility vs. no visibility). We assessed four dependent variables: Affect rating, question accuracy, question response time (RT; in correctly answered trials), and prosociality rating (in Exps. 1, 2 and 4). These analyses deviate from our initial plan (see, e.g., preregistration 10.17605/OSF.IO/DS4VE) which proposed conducting separate analyses of variance (ANOVA) for each experiment and then pooling the data for combined LMMs. In retrospect, we determined that computing experiment-wise LMMs would be a more robust approach, allowing us to maximize power and better account for variability in the data. However, we also report the originally planned analyses in Supplement S3. Notably, results across the different analysis approaches were largely consistent and did not alter our conclusions.

Our analysis focused on several key effects that were central to addressing our research question: An impairment of empathic responding would be reflected in a reduced affect rating difference between neutral and negative narrations when faces were (partially) covered. Statistically, this would manifest as a significant interaction between Visibility and Valence while the main effect of Visibility – reflecting general differences in mood – was not directly relevant to our hypotheses. For accuracy and RTs in the single-choice questions, a significant main effect of Visibility would suggest a general impairment of understanding when the full face was not visible. A significant interaction between Visibility and ToM Requirement, on the other hand, could indicate specific difficulties related to ToM (or noToM). Finally, for prosociality rating, we examined both the main effect of Visibility (indicating overall reduced willingness to help), as well as the interaction between Visibility and Valence (specifically addressing prosocial response towards individuals in need).

### Affect rating

**Fig. 2:**
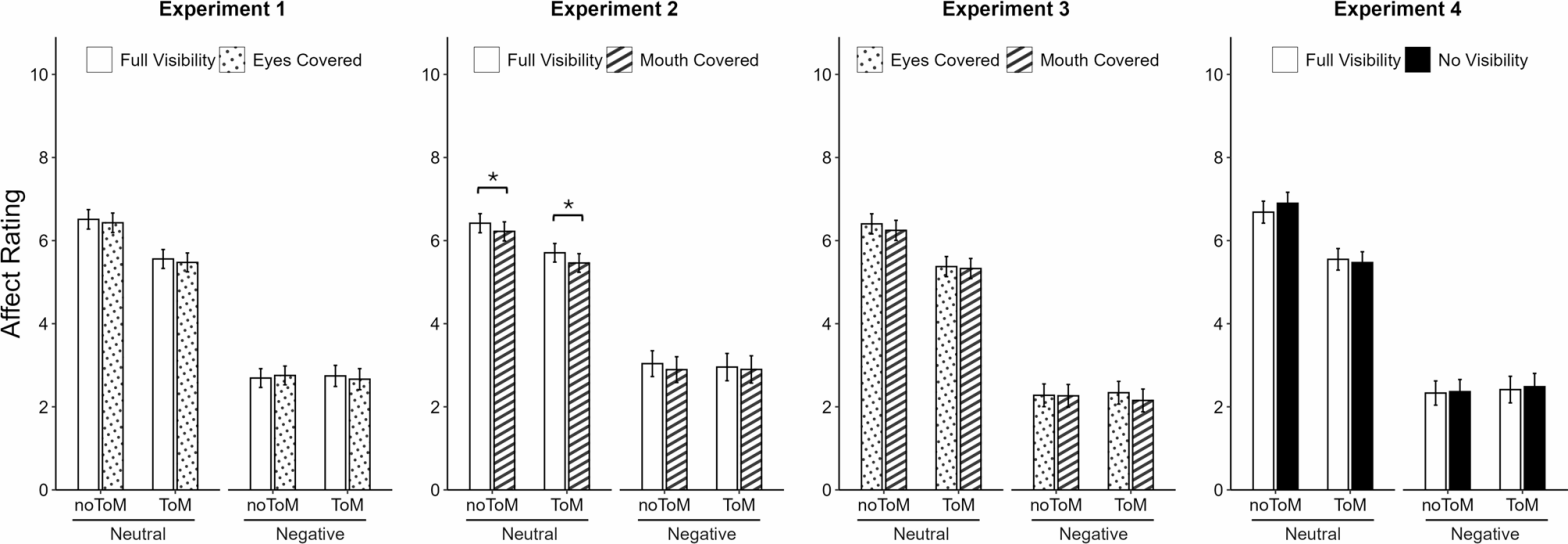
Estimated marginal means for affect rating in Experiments 1–4. Differences between the rated affect after negative versus neutral narrations were interpreted as empathic responding. Error bars represent standard errors. On the x-axis, the different combinations of Valence and ToM Requirement are displayed. Bar colors and patterns denote Visibility condition (full visibility: white; eyes covered: dots; mouth covered: diagonal stripes; no visibility: black). Significant differences between Visibility conditions within each Valence x ToM Requirement combination are marked with horizontal brackets. *: *p* <.05.

Estimated marginal means for affect ratings (on a scale from 0 to 10, with symbolic anchors at the endpoints and midpoint) are displayed in Fig. 2. In addition to the random intercepts for participants and narrations, the final models for Experiments 1, 2, and 4 included random slopes for Valence and ToM Requirement, whereas the final model for Experiment 3 only featured a random slope for Valence. Note that the computation of these models deviated from the preregistered plan (see 4.4 ‘Design and Analyses’). Across the four experiments, participants reported lower affect following negative narrations compared to neutral ones (estimated marginal means Exp.1: 2.71 vs. 5.99; Exp.2: 2.95 vs. 5.95; Exp.3: 2.26 vs. 5.84; Exp.4: 2.40 vs. 6.15), confirming the effectiveness of our valence manipulation and replicating previous findings from the original task^49,64,65,74^, *b*s < −3.01, *t*s < −10.63, *p*s < 0.001. While affect ratings did not differ between ToM conditions after negative narrations (*t*s < |0.32|, *p*s > 0.753, *d*s < |0.06|), participants rated their affect slightly lower after neutral ToM compared to neutral noToM narrations (*t*s > 2.73, *p*s < 0.009, *d*s > 0.52), as indicated by significant (or in the case of Exp. 2, marginally significant) Valence x ToM Requirement interactions, *b*s > 0.69, *t*s > 1.86, *p*s < 0.068, and main effects of ToM Requirement, *b*s < −0.38, *t*s < −1.99, *p*s < 0.052. While they are not central to our research questions, these patterns also replicate earlier findings^49,65^.

For the critical Visibility x Valence interaction – reflecting a potential moderation of empathic responses by narrator visibility – no significant effect was observed in any of the experiments, *b*s < |0.12|, *t*s < |1.25|, *p*s > 0.210. Hence, what part of the narrators’ faces were visible did not significantly influence participants’ empathy with the narrators. The main effect of Visibility was significant in Experiment 2, *b* = −0.16, *t*s = −3.33, *p*s < 0.001, but not in Experiment 3, *b* = −0.10, *t* = −1.86, *p* = 0.063, or in Experiments 1 and 4, *b*s < |0.06|, *t*s < |1.09|, *p*s > 0.274: Participants rated their affective state slightly lower overall when the narrator’s mouth was covered compared to when the face was fully visible (Exp.2 – mouth covered: 4.37 vs. full visibility: 4.53; Exp.3 numerical difference: mouth covered: 4.00 vs. eyes covered 4.10). No further main effects or interactions reached significance, *b*s < |0.33|, *t*s < |1.49|, ps > 0.136.

### Question accuracy

**Fig. 3:**
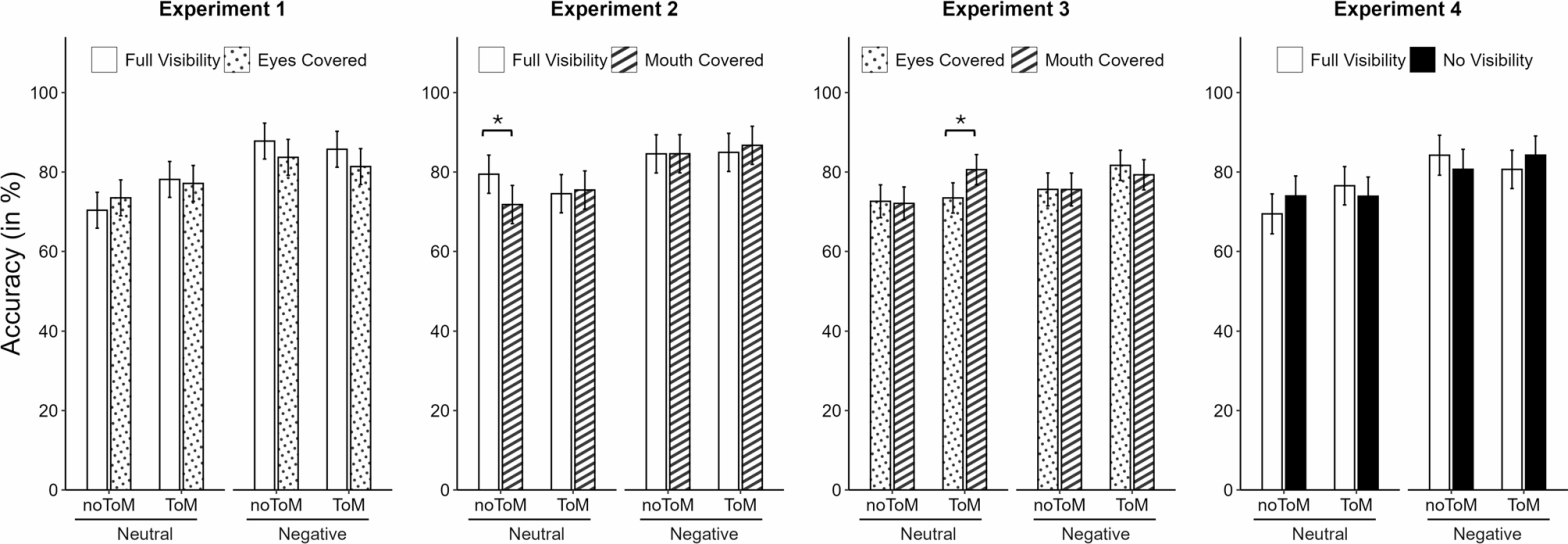
Estimated marginal means for question accuracy in Experiments 1–4. Error bars represent standard errors. On the x-axis, the different combinations of Valence and ToM Requirement are displayed. Bar colors and patterns denote Visibility condition (full visibility: white; eyes covered: dots; mouth covered: diagonal stripes; no visibility: black). Significant differences between Visibility conditions within each Valence x ToM Requirement combination are marked with horizontal brackets. *: *p* <.05.

Estimated marginal means for question accuracy (in %) are presented in Fig. 3. The final models (computed in deviation from the preregistered plan, see above) for Experiments 1 and 2 did not feature any random slopes, while for Experiments 3 and 4, the best-fitting models included random slopes for ToM Requirement. Accuracy was slightly higher for ToM questions than for noToM questions (Exp.1: 80.6% vs. 78.8%; Exp.2: 80.4% vs. 80.1%; Exp.3: 78.8% vs. 74.0%; Exp.4: 78.8% vs. 77.1%), although the difference did not reach statistical significance in any experiment, *b*s < 0.05, *t*s < 1.31, *p*s > 0.196. In Experiments 1, 2, and 4, questions following negative narrations were answered with higher accuracy compared to neutral narrations as reflected in significant main effects of Valence, *b*s > 0.09, *t*s > 2.06, *p*s < 0.045 (Exp.1: 84.6% vs. 74.8%; Exp.2: 85.2% vs. 75.3%; Exp.4: 82.5% vs. 73.5%). In Experiment 4, the significant three-way interaction, *b* = 0.14, *t* = 2.02, *p*s = 0.044, indicated that the valence effect was stronger in noToM trials with fully visible narrators (*t*(64.9) = −2.22, *p* = 0.030, *d* = −0.48) compared to the other Visibility x ToM Requirement combinations (*t*s < |1.56|, *p*s > 0.125, *d*s > |0.35|).

Most importantly for our hypotheses, there were no significant main effects of Visibility, *b*s < |0.02|, *t*s < |0.91|, *p*s > 0.365, and Visibility x ToM Requirement interactions, *b*s < |0.05|, *t*s < |1.55|, *p*s > 0.122, in any of the experiments. Thus, we found no evidence that mentalizing performance was affected by which part of the narrators’ faces participants could see. No other main effects or interactions reached significance, *b*s < |0.60|, *t*s < |1.51|, *p*s > 0.131.

### Question response time

As a complementary analysis, we also examined the time it took participants to give correct responses in the single-choice questions, although interpretability is limited due overall long response durations (which included reading time) and the emphasis on accuracy over speed in the instructions. The best-fitting models for RTs (in seconds) did not include random slopes but only the random intercepts for participant and narration. Analyses of these models revealed one significant effect, which was the interaction between Visibility and ToM Requirement in Experiment 2, *b* = 0.91, *t* = 2.15, *p* = 0.032. It took participants slightly longer to respond to ToM questions when the narrator’s mouth was covered compared to fully visible (16.8 s vs. 16.3 s; marginally significant: *t*(1424) = −1.89, *p* = 0.059, *d* = −0.13), while there was no comparable difference for noToM questions (16.0 s vs. 16.4 s, *t*(1424) = 1.15, *p* = 0.249, *d* = 0.08). Apart from that, no further main effects of Visibility or interactions between Visibility and ToM Requirement reached significance, *b*s < |0.33|, *t*s < |1.24|, *p*s > 0.215. The same applied to all remaining main effects and interactions, *b*s < |2.38|, *t*s < |1.98|, *p*s > 0.053.

### Prosociality rating

**Fig. 4:**
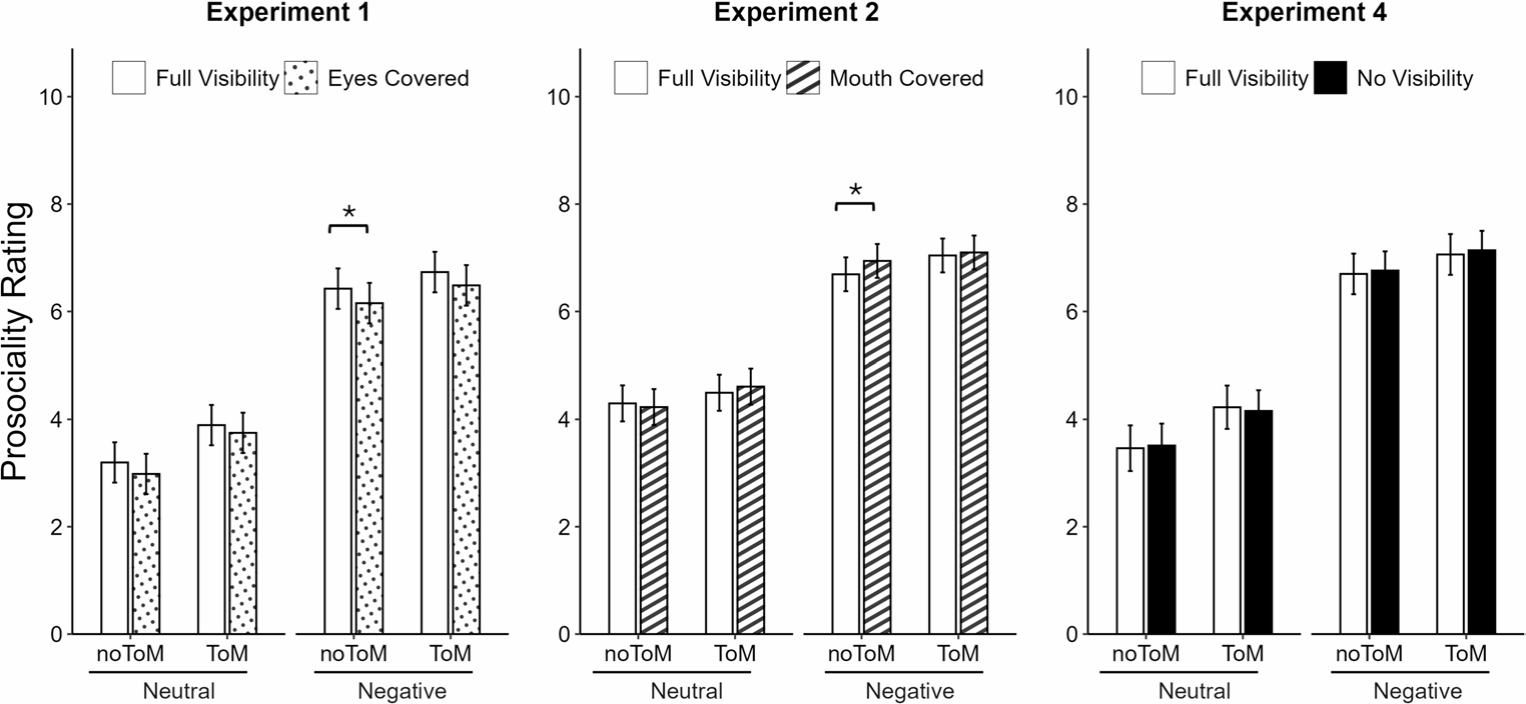
Estimated marginal means for prosociality rating in Experiments 1, 2, and 4. Error bars represent standard errors. On the x-axis, the different combinations of Valence and ToM Requirement are displayed. Bar colors and patterns denote Visibility condition (full visibility: white; eyes covered: dots; mouth covered: diagonal stripes; no visibility: black). Significant differences between Visibility conditions within each Valence x ToM Requirement combination are marked with horizontal brackets. *: *p* <.05.

Estimated marginal means for prosociality ratings (on a scale from 0 to 10) in the three experiments that measured this dependent variable (Exps. 1, 2, 4) are shown in Fig. 4. The final models (derived from analyses deviating from the preregistration, see above) for Experiments 1 and 2 included random slopes for Valence, while for Experiment 4, incorporating random slopes for all three factors (Visibility, Valence, ToM Requirement) led to the best model fit. In line with earlier findings^63,74^, a significant main effect of Valence indicated that participants were willing to invest more resources to help a narrator recounting a negative experience compared to a neutral one (Exp.1: 6.45 vs. 3.46; Exp.2: 6.94 vs. 4.41; Exp.4: 6.92 vs. 3.84), *b*s > 2.53, *t*s > 7.42, *p*s < 0.001. Additionally, in Experiment 1, prosociality ratings were significantly higher following ToM compared to noToM narrations (5.21 vs. 4.69), *b* = 0.53, *t* = 2.06, *p* = 0.045.

The main effect of Visibility, which was central to our hypotheses, reached significance in Experiment 1, *b* = −0.22, *t* = −3.21, *p* = 0.001: Participants were less willing to invest resources to help a person whose eyes were covered compared to when they were fully visible (4.84 vs. 5.06). In Experiments 2 and 4, on the other hand, visibility had no effect on prosociality, *b*s < 0.09, *t*s < 1.46, *p*s > 0.143, indicating that impaired visibility of narrators’ mouths (Exp.2) or complete faces (Exp.4) did not significantly reduce participants’ willingness to invest resources. The remaining main effects and interactions did not reach statistical significance *b*s < |0.54|, *t*s < |1.90|, *p*s > 0.063, including the interaction between Visibility and Valence, *b*s < |0.13|, *t*s < |1.09|, *p*s > 0.278.

To explore the relationship between empathizing, mentalizing, and willingness to help, we computed correlations between affect ratings und prosociality ratings as well as between accuracy and prosociality ratings on the interindividual level across Experiments 1, 2, and 4. For negatively valenced narrations, we observed a strong negative correlation between affect ratings and prosociality ratings, *r* = −0.42, *p* < 0.001, while no significant correlation was found in the neutral condition, *r* = 0.10, *p* = 0.290. This suggests that participants were more willing to invest (hypothetical) resources if they empathized strongly with a narrator recounting a negative experience. The correlation between accuracy and prosociality did not reach significance for either ToM questions, *r* = 0.16, *p* = 0.080, or noToM questions, *r* = 0.03, *p* = 0.731.

### Bayes factors

Given that our LMM analyses revealed mostly null effects for the key comparisons relevant to our hypotheses, we conducted additional Bayesian analyses to quantify the strength of evidence for the absence of effects. Specifically, we performed Bayesian *t*-tests on the raw data (separate from the LMM framework) to compute Bayes Factors in favor of the null hypothesis (BF₀₁), using a standard Cauchy prior of 0.707.

For empathic responding, we calculated the difference in affect ratings between negative and neutral narrations within each visibility condition and then compared these differences across conditions. According to common classifications of Bayes Factor^75,76^ all four experiments provided moderate support for the null hypothesis (Exp. 1: BF₀₁ = 4.06; Exp. 2: BF₀₁ = 3.59; Exp. 3: BF₀₁ = 5.88; Exp. 4: BF₀₁ = 5.59).

For accuracy, Bayesian *t*-tests again indicated moderate evidence for no difference in performance between visibility conditions (Exp. 1: BF₀₁ = 4.63; Exp. 2: BF₀₁ = 4.97; Exp. 3: BF₀₁ = 5.41; Exp. 4: BF₀₁ = 5.69). With respect to the interaction between Visibility and ToM Requirement on accuracy, Bayes Factors provided moderate support for the null in all experiments, with the exception of Experiment 2, which showed only anecdotal evidence (Exp. 1: BF₀₁ = 4.55; Exp. 2: BF₀₁ = 1.52; Exp. 3: BF₀₁ = 4.43; Exp. 4: BF₀₁ = 5.73).

For prosociality, the significant visibility effect observed in the LMM for Experiment 1 was accompanied by anecdotal evidence for the alternative hypothesis (BF₀₁ = 0.45). In contrast, Experiments 2 and 4 yielded anecdotal to moderate evidence for the null (Exp. 2: BF₀₁ = 2.07; Exp. 4: BF₀₁ = 5.09). Finally, for the interaction between Visibility and Valence on prosociality, all relevant comparisons provided moderate support for the null hypothesis (Exp. 1: BF₀₁ = 5.63; Exp. 2: BF₀₁ = 4.23; Exp. 4: BF₀₁ = 5.43).

## Discussion

While the challenge of interacting with individuals whose faces are (partly) covered is not new, the widespread use of protective masks during the Covid-19 pandemic has intensified discussions about the impact of reduced face visibility. In this context, numerous studies have reported impaired emotion recognition when parts of the face were occluded^9,11,14,15^. The goal for our study was to investigate whether similar detrimental effects would emerge for more complex aspects of social understanding – specifically, empathizing and mentalizing – as well as social decision making when provided with realistic and contextual cues that might enable compensation for the reduced visual information^41,42,77^. In a video-based paradigm^65^, we presented short autobiographical narrations of either neutral or negative valence, followed by questions about the participant’s affective state, understanding, and willingness to help. The clips were manipulated to show the narrator’s face in different visibility conditions: fully visible, with black bars covering either their eyes or mouth, or with only the narrator’s voice audible.

Across four experiments, consistent patterns emerged for all variables of interest. Firstly, the *affect ratings* provided by the participants after each narration showed that our stimuli successfully elicited empathic responding, in line with previous studies using this task^64,65,78^. Interestingly, participants reported slightly lower overall affect when the narrator’s mouth was covered. However, the effect of narration valence – i.e., the difference in affect between neutral and negative narrations – did not significantly vary between visibility conditions. Hence, critical to our main hypothesis, the extent of empathic responding was not influenced by whether participants could see the narrator’s full face, their upper face, their lower face, or nothing at all.

Secondly, participants generally performed well on questions about the narrations’ content, achieving a mean accuracy of around 80% both for *mentalizing* and factual reasoning. This finding aligns with the accuracy levels reported in prior studies^49,65,74^ and suggests that ceiling effects are unlikely to affect our results. Importantly, accuracy rates did not indicate any disadvantage for mentalizing performance when the narrator’s face was partially or fully obscured. We additionally analyzed response times for correct answers, though this provides complementary rather than central insights as response times were relatively high (due to the time required to read the question) and instructions prioritized accuracy over speed. Similar to accuracy, response times were not substantially affected by the visibility manipulation, with a single exception: In one of the experiments, participants took slightly longer to answer Theory of Mind questions when the narrator’s mouth was covered versus fully visible, while no such delay emerged for factual reasoning questions.

Finally, *prosociality* was – in line with Lehmann et al.^63^ – associated with empathic responding: Participants who reported lower affect after negative narrations were more willing to help the narrator compared to participants whose affect was less diminished while we did not observe a significant correlation between accuracy and prosociality ratings. Our main analysis revealed that participants were more willing to (hypothetically) invest resources to help narrators who recounted a negative autobiographical story compared to a neutral one, as in previous studies^63,74^. This valence effect remained consistent across visibility conditions, indicating that the increase in prosociality after learning about someone’s bad experiences (compared to neutral experiences) was not significantly affected by facial feature visibility. However, overall prosociality – irrespective of emotional valence – was lower when the narrators’ eyes were covered compared to when their faces were fully visible. Since no similar effects were observed when the mouth or the full face (including the eyes) was covered, this likely does not reflect a general reluctance to help individuals whose faces are not fully visible. Instead, it may point to more specific effects for covered eyes or even the particular type of coverage used in the study (as we discuss later).

Integrating all these results, we found that participants’ social understanding (i.e., empathy and ToM) was not substantially impaired by limited visibility of the speaker’s face, as levels of empathizing and mentalizing were maintained across conditions in all four experiments. Perhaps most strikingly, even removing visual input completely in one of our experiments did not significantly impair social understanding compared to unobscured face visibility. This contrasts with prior findings on the detrimental effects of (partial) face coverage^4,7,9^ as well as the differential importance of eye and mouth visibility for emotion recognition^18,20,21^. It is apparent that participants in our study utilized other cues and modalities to compensate for the reduced visibility of the narrator. For example, when the narrator’s mouth or eyes were covered, participants might have instead concentrated on other areas of the face or on body posture^41^. However, since even these signals were unavailable in the no visibility condition, the narrator’s voice likely served as the most important source of information, both non-verbal and verbal. Numerous prior studies have documented humans’ ability to interpret tone of voice and other vocal cues, observing high levels of emotion recognition even in the absence of accompanying visual information^31,48^. Additionally, the verbal content of our study’s narrations offered extensive contextual information about the narrator’ affective state. As demonstrated by McCrackin and Ristic^77^, providing even a single line of context can significantly mitigate the negative impact of facial coverage. It therefore seems plausible that in our study, the narrator’s voice provided sufficient information to compensate for the lack of facial cues. While it is possible that part of this compensation was a voluntary effort by participants to give socially desirable answers for the affect and prosociality ratings, critically, the same is impossible for mentalizing given the performance-based nature of its assessment. Consequently, our data provide substantial support for the possibility of unimpaired empathizing and mentalizing, even when the interaction partner’s face is partially or fully covered. The dynamic and multimodal nature of our stimuli likely played a pivotal role in shaping these findings. While studies using static and artificial stimuli have overwhelmingly reported detrimental effects of face masks, research employing more naturalistic procedures has found no significant impact on empathy and connectedness^42,43^. Our results align well with these latter findings, which more closely represent real-life interactions, thereby underscoring the importance of ecological validity when studying social cognition.

Even though these findings are encouraging in terms of the potential for compensation in less-than-ideal circumstances, it would certainly be an overreach to conclude that (partial) face coverage does not have any adverse effects on social interactions. In fact, there seems to be some evidence for this in our own data. First, there is the decrease in prosociality towards individuals whose eyes were not visible, as observed in one of the experiments. Participants may have felt less closeness and connectedness to these narrators, especially given the cover story that they intentionally wanted their identity to be concealed. This effect may have been further intensified by our choice of “neutral” coverage using black bars: In everyday contexts, black bars over the eyes are often associated with anonymizing individuals in criminal investigations. If this imagery evoked associations with untrustworthy or suspicious people, it could explain why participants were less willing to help the narrators in this condition. Alternatively, the decrease in prosociality may reflect that in the eyes covered condition, participants felt less watched and, consequently, were less self-aware and concerned about social expectations^79–81^. To distinguish between these potential explanations (dependent either on the coverage itself or the specific type of coverage) it could be helpful to replicate our experiment using a more naturalistic form of coverage (e.g., adding sunglasses to the narrators’ faces) in the future. Another potential detrimental effect of face coverage is that while people may not be objectively worse at understanding their interaction partner when they cannot see their full face, they might still find it less pleasant or more effortful^82^. This could explain the slightly more negative affect reported by participants when the narrator’s mouth area was covered as well as participants taking slightly more time to think about questions requiring Theory of Mind (while giving equally accurate responses) in one of the experiments. Interestingly, a similar discrepancy between objective performance and subjective judgments has been reported in previous studies investigating the effect of masks on emotion recognition^41,83^. For example, Ross and George^41^ found that participants were less confident in their emotion recognition performance for masked faces, despite no actual decrease in accuracy. Additionally, the face occlusion in our experiments might have been perceived as a sign of unapproachability, disconnectedness or untrustworthiness, contributing to the negative affective response^11,82,84^.

It is also important to keep in mind that our experimental setup in many ways provided optimal circumstances for compensation. Even though our task is quite challenging, as narrators recount relatively complex autobiographical stories in a short time period (12–15 s), the audio tracks offered contextual information, delivered with articulatory clarity and high-quality sound. In contrast, real face masks create a physical barrier that can occasionally impede not only visibility but also clarity of speech^82,85,86^. In addition, there were no other cues vying for the participants’ attention in our paradigm, such as background noises or additional tasks. As most people can probably attest, it is notably more challenging to discern another person’s inner workings when they are squeezed next to you in a crowded subway than when you are sitting in the peace of your living room. It is therefore plausible that the ability to compensate for missing information reaches a limit when environmental factors are disadvantageous and attentional resources are otherwise engaged. The specific threshold for this limit could also vary between individuals. For example, persons with sensory impairments or symptoms of dementia are likely to be more severely affected by reduced visual information^8,82,87,88^. Exploring whether and how our pattern of results might change under particularly challenging conditions, such as additional distractions, could further enhance understanding of the underlying processes.

Another aspect potentially influencing our results might be the respective comparison conditions of visibility in each experiment. Since practical constraints (such as maintaining sufficient trials per condition) prevented us from comparing all four visibility conditions in a single within-subject experiment, we cannot rule out that the stimuli were evaluated differently depending on the condition they were contrasted against. For instance, covering the eyes might be perceived as an obstructive blocking of information when contrasted with fully visible faces but less so when compared to faces where other regions are occluded in a similar manner. This effect might have been exacerbated by the instructions framing the different types of coverage with distinct justifications (e.g., anonymizing narrators, investigating language comprehension) which potentially introduced motivational or emotional biases. A related limitation applies to our emotional valence conditions, which contrasted only neutral and negative narrations. The omission of other valence conditions (such as positive narrations) may have created specific contrast effects that do not fully represent the broader pattern of empathic responding, particularly positive empathy. There might have also been contrast effects *within* the experiment, e.g., when multiple trials of the same condition were presented consecutively, although exploratory analyses did not suggest that carry-over effects significantly affected our main results (see Supplement S4).

Despite the limitations discussed above, we believe that – considering the four experiments in combination – our results clearly demonstrate the human ability to flexibly compensate when visual information in social interactions is reduced. Without explicitly delving into evolutionary theories, it seems intuitively plausible that humans, at some point, developed strategies to cope with limited facial information. Such adaptations would have been crucial for effective communication with conspecifics over distances, at night, or in densely vegetated areas, even without considering artificial obstacles like brick walls and sunglasses. We believe that acknowledging this potential for compensation could shift the focus from solely emphasizing impairments in split-second emotion recognition, towards exploring social understanding in conditions that more closely reflect the complex, dynamic, and multimodal nature of naturalistic interactions^27,89–91^. A better insight into the mechanisms and prerequisites for successful compensation could, in turn, help create conditions that facilitate it, particularly in interactions where accurate understanding and empathizing are essential. For instance, in a clinical setting where masks are indispensable, this could involve setting up a calm, distraction-free space for discussing sensitive topics. Or, in everyday interactions like talking to a co-driver, it might simply mean being careful to provide ample verbal context when making arrangements without visual contact to avoid misunderstandings.

In essence, our findings demonstrate robust empathizing and mentalizing even when an interaction partner’s face is partially or fully occluded. This suggests that participants in our study effectively compensated for the missing facial information by relying on other available signals, particularly vocal cues. We observed a slight reduction in prosociality when the interaction partner’s eyes were covered (but not for mouth or full-face occlusion), possibly hinting at effects of specific types of coverage on social decision making but not suggesting a general reduction in willingness to help when facial visibility is reduced. Taken together, we argue that it is important to acknowledge not only the adverse effects of masks and other visual barriers but also the adaptive abilities that can mitigate these challenges in naturalistic settings. Adopting this balanced perspective would help to promote a more comprehensive view of social understanding^49,92,93^, one that reflects the remarkable flexibility inherent in human social cognition.

## Methods

### Sample

A total of 165 individuals participated in one of four experiments that took place between September 2021 and October 2022. Recruitment was conducted through university online platforms (University of Hannover, University of Würzburg), online posts, and private contacts. Data from eight participants were excluded from the analyses because their error rates were more than two standard deviations above the mean of their respective experiment. Consequently, the final sample sizes for the experiments ranged from 38 to 41 participants (total *N* = 157). The mean sample age varied from 21.6 to 27.1 years, with female participants representing between 70.7% and 81.6% of the samples. Table 1 summarizes demographic characteristics for Experiments 1–4. Participants received either monetary compensation, course credit, or bookstore vouchers for their participation. The procedure complied with the ethical standards of the 1964 Declaration of Helsinki regarding the treatment of human participants in research. The paradigm employed in this study (EmpaToM task) has been approved by ethics committees (Ethikkommission des Institutes für Psychologie der Humanwissenschaftlichen Fakultät der Julius-Maximilians-Universität Würzburg, GZEK 2017-04).

### Stimulus material

In the course of the study, we presented video clips of different narrators recounting autobiographical episodes, which were taken and adapted from the validated EmpaToM paradigm^65^. Each video clip lasted approx. 15 s and featured a male or female individual whose face and upper body were shown from a frontal perspective in 4:3 format. The narrations dealt either with neutral experiences, such as work and leisure activities, or emotionally negative events, such as losing a loved one or getting injured (manipulation of Valence: *neutral* vs. *negative*). A total of 12 narrators (six female and six male, of different age groups) were selected for the study. Each narrator was featured in four videos, two neutral and two negatively valenced. Within each valence condition, one narration gave rise to a question that required mentalizing (e.g., recognizing ironic subtext, reading “between the lines”) while the other involved purely factual reasoning (manipulation of ToM Requirement: *noToM* vs. *ToM*). Two sets of narrations and associated questions are provided as examples in the supplements (Supplement S1).

The EmpaToM videos were displayed unaltered in the full visibility condition, while modifications were made for three additional conditions, varying which parts of the narrator’s face were visible to the participant (manipulation of Visibility: *full visibility* vs. *eyes covered* vs. *mouth covered* vs. *no visibility*). In the eyes covered and mouth covered condition, we added black bars, approximately 1/4th of the video’s width and 1/7th of the video’s height, and placed them over either the eye or the mouth area, respectively. Throughout each clip, the bar remained stationary and did not follow the narrators’ head movements. However, the relevant region (eye area/mouth area) was concealed at all times. In the no visibility condition, we isolated the audio from each video and played it over a blank screen. Each participant encountered all clips from one narrator in the same Visibility condition, with the assignment of narrators to conditions counterbalanced between participants.

### Procedure

The experiment was designed and conducted with PsychoPy^94^. Data collection took place at the Julius-Maximilians-Universität Würzburg (Exps. 1, 2, 4) and the Leibniz Universität Hannover (Exp.3), with up to four individuals participating simultaneously. After giving their informed consent, participants were seated in front of a computer monitor with a standard German keyboard and over-ear headphones. They received both written and verbal instructions about the experimental procedure. Depending on the specific experiment, different cover stories were provided to explain the Visibility conditions. For the eyes covered condition, participants were told that a bar was placed over the eye area, because the narrators in the respective video wished to remain anonymous (Exps. 1 and 3). The feigned reason for covering the mouth was either to test the dependence of language comprehension on mouth visibility (Exp.2) or to create a control condition for the narrators with covered eyes (Exp.3). Finally, it was explained that the audios in the no visibility condition were sourced from earlier studies, with video footage available only for the “more recently recorded” narrations (Exp.4).

To familiarize themselves with the task, participants completed two practice trials. After confirming that there were no remaining questions, they proceeded to complete 48 trials in randomized order. During these trials, each participant encountered all possible narrations in one of the two visibility conditions included in their experiment. The study took about 40 min, with a break after half of the trials. Each trial began with a fixation cross (2 s), followed by the narrator’s name (2 s), and then the narration (15 s) presented in the respective Visibility, Valence, and ToM Requirement condition (a schematic depiction of the experimental procedure is provided in Fig. 1; the individual in the example images provided informed consent for their publication in an online open-access publication). After each clip, participants were first asked to rate their own affective state (*affect rating*: “How are you feeling?”, in German: “Wie fühlst du dich?”) on a scale with three marked anchors: A sad emoji face at the left end, a neutral face in the center, and a happy face at the right end. Participants used the arrow keys on the keyboard to move the marker across the scale in increments of one-tenth of the scale length and confirmed their selection by pressing Enter. After that, another fixation cross appeared, followed by a single-choice question about the content of the narration with three response options. In the noToM condition, the questions started with “It is true that…” (in German: “Es stimmt, dass…”), while in the ToM condition, questions were prefaced by “[Name] thinks that…” (in German: “[Name] denkt, dass…”). Participants were required to press one of three marked keys to select the option which they believed to be a correct statement based on the presented narration. They were given no time restriction and instructed to work conscientiously rather than quickly. Accuracy and RTs for correct responses were collected as measures of the participants’ ToM and factual reasoning performance (question accuracy, question RT). While the trial ended at this point in Experiment 3, all other experiments additionally recorded a rating for prosociality (preceded by another fixation cross). For this last rating, participants were asked to indicate their willingness to help the narrator (prosociality rating: “How much resources would you be willing to invest to help the person?”, German: “Wie viele deiner Ressourcen würdest du investieren, um der Person zu helfen?”) on a 10-point rating scale. The ends of the scale were labeled “nothing at all” and “a lot” (German: “gar nichts” – “sehr viel”), from left to right. In the initial instructions, it was emphasized that “resources” could refer to financial as well as emotional or practical support, depending on the narrator’s needs. After completing the trials, participants filled out a questionnaire that collected their demographic data, asked them what they thought the objective of the study was, and gave them the opportunity to leave additional comments about any issues that occurred during the experiment. Participants were then debriefed and thanked for their participation.

### Design and analysis

We chose to divide our study into four experiments to avoid an overly large number of trials and to reduce potential suspicions regarding the experimental manipulation. Each experiment compared two of the overall four visibility conditions: Exp. 1: full visibility vs. eyes covered; Exp. 2: full visibility vs. mouth covered; Exp. 3: eyes covered vs. mouth covered; Exp. 4: full visibility vs. no visibility (audio-only). Combined with the Valence (neutral vs. negative) and ToM Requirement (noToM vs. noToM) manipulation of the EmpaToM, this resulted in a 2 × 2 × 2 within-subjects design per experiment. We conducted separate analyses for each experiment, applying linear mixed models (LMMs). Our initial analysis plan (see e.g., preregistration 10.17605/OSF.IO/DS4VE) had been to calculate repeated-measures ANOVAs for each experiment and to subsequently pool the data from multiple experiments to compute combined LMMs. The results of these planned analyses are available in the supplements (Supplement S3). However, to enhance statistical power and better account for variance associated with different participants and stimuli, we ultimately decided to apply LMMs (instead of ANOVAs) to the data of each experiment. While we did not have specific hypotheses regarding the random effects, their inclusion allowed us to account for structured variability in our data, thereby improving model fit and reducing unexplained noise. Note, however, that the overall pattern of results remained consistent across the different analytical approaches and supported the same conclusions.

Analyses were conducted for four dependent variables: (1) *Affect rating* (0–10 on a scale with ten increments where the midpoints and endpoints were anchored), where we were mainly interested in the effect of face visibility on empathic responding, as reflected in the difference between affect in neutral and negative trials (i.e., the Visibility x Valence interaction). (2) *Question accuracy* (in %), where differences could be observed specifically for ToM questions (reflected in a Visibility x ToM Requirement interaction) or for overall understanding. (3) *Question RT* (in seconds), with a similar focus as accuracy. For RT analyses, we included only trials with correct responses and additionally removed those with exceptionally high RTs, defined as more the 3 SDs above the experiment mean (1.5% of the trials with correct responses). (4) *Prosociality rating* (on a scale from 0 to 10), examining both the overall effect of Visibility as well as its interaction with Valence. Prosociality was only assessed in Experiments 1, 2 and 4.

For each of these dependent variables we fitted LMMs to the trial-wise data per experiment, employing Maximum Likelihood estimation and Satterthwaite approximation for the degrees of freedom (packages ‘lme4’^95^ and ‘lmerTest’^96^ in R, version 4.3.1^97^). The LMMs included Level 1 fixed effects for Valence (coded as neutral = −0.5, negative = 0.5) and ToM Requirement (noToM = −0.5, ToM = 0.5). Additionally, we modeled Visibility as a Level 1 factor (Exp.1: full visibility = −0.5, eyes covered = 0.5; Exp.2: full visibility = −0.5, mouth covered = 0.5; Exp.3: eyes covered = −0.5, mouth covered = 0.5; Exp.4: full visibility = −0.5, no visibility = 0.5).

Each model began with a baseline structure including only the fixed effects and random intercepts for participants and narrations. We then incrementally added random slopes for Visibility, Valence, and ToM Requirement by participant and conducted Likelihood Ratio Tests for model comparison. Only random slopes that significantly improved model fit without causing singularity issues were retained. In the Results section, we report findings from the best-fitting model for each dependent variable and experiment. To ensure robustness, we also conducted supplementary analyses using both a minimal model (random intercepts only) and a maximal model (including all random slopes, as recommended by^98^). These alternative models produced no meaningful differences in results. Full details of all LMMs are available in the supplements (Supplement S2).

In the Results section, we report unstandardized coefficients (*b*) and the associated *t*- and *p*- values as calculated by the ‘lmerTest’^96^ package in R. For post-hoc comparisons, we provide estimated marginal means and *t*-tests computed with the ’emmeans’^99^ package. Cohen’s *d* was estimated based on the resulting *t*-value, sample size and the correlation between the repeated measures^100^.

In addition to our primary analyses, we conducted supplementary Bayesian analyses for the comparisons most relevant to our hypotheses. Specifically, we performed paired-samples Bayesian *t*-tests on the raw (unmodeled) data to compute Bayes Factors (BF₀₁), which quantify the likelihood of the data under the null hypothesis relative to the alternative. All Bayesian analyses were conducted using the R package ‘BayesFactor’^101^, with the default Cauchy prior of 0.707. We interpreted the resulting Bayes Factors following standard classifications^75,76^, with values of BF₀₁ between 3 and 10 indicating moderate evidence for the null. These analyses were applied to the key comparisons for affect rating (interaction Visibility x Valence), accuracy (main effect Visibility, interaction Visibility x ToM Requirement), and prosociality (main effect Visibility, interaction Visibility x Valence) across all experiments.

To investigate interindividual differences in the association between empathy and prosociality, we computed Pearson correlations between the participant-wise means for affect rating and prosociality rating, separated by valence condition (Exps. 1, 2, 4). Similarly, we examined the correlation between question accuracy and prosociality rating – separately for the ToM and noToM condition – to explore the potential association between accurate mentalizing and prosociality.

## Electronic supplementary material

Below is the link to the electronic supplementary material.

Supplementary Material 1

## Data Availability

The raw data and the R script used for the analyses are available on the Open Science Framework repository (https://osf.io/ndbwy/?view_only=42ab4f9690634eb78032d8c648372113).
