# Supplementary material for: No evidence for disruption of empathy and mentalizing by face coverage in multimodal settings
Source: Sci Rep. 2025 Dec 5;15:43281. doi: 10.1038/s41598-025-25393-7 (PMC12686505; doi:10.1038/s41598-025-25393-7)
Supplement: Supplementary file 1 — Supplementary Material 1 [file 41598_2025_25393_MOESM1_ESM.pdf]

Supplementary Materials for

No evidence for disruption of empathy and mentalizing by face coverage in multimodal settings

Eva Landmann[1*], **Inga Felicia Straub**[1], and **Anne Böckler**[1]

[1]Julius-Maximilians-Universität Würzburg, Department of Psychology, Würzburg, 97070, Germany
*eva.landmann@uni-wuerzburg.de

Content:

S1 Example Narrations

S2 Supplementary Material for Main Analyses

S2.1 Full LMM Tables

S2.2 Estimated Marginal Means

S3 Originally Planned Analyses

S3.1 Analyses of Variance

S3.2.1 Methods
S3.2.2 Results
S3.2.3 Supplementary Tables

S3.2 Linear Mixed Models on Pooled Data

S3.3.1 Methods
S3.3.2 Results
S3.3.3 Supplementary Tables

S4 Exploratory Analyses of the Influence of Previous Trials

S1 Example Narrations

In our study, we adapted video stimuli from the EmpaToM paradigm (Kanske et al., 2015). We utilized sets of 12 narrators, each presenting 4 narrations. These narrations varied in emotional valence (neutral vs. negative) and in the type of question they prompted (ToM Requirement: noToM vs. ToM). The following two example sets were translated into English from their original German versions by the authors. Correct answers to the corresponding questions are underlined.

Kanske, P., Böckler, A., Trautwein, F.-M., & Singer, T. (2015). Dissecting the social brain: Introducing the EmpaToM to reveal distinct neural networks and brain–behavior relations for empathy and Theory of Mind. *NeuroImage*, *122*, 6–19. https://doi.org/10.1016/j.neuroimage.2015.07.082

Hannes (23 years old)

Narration 1 - Neutral & noToM

"I am currently doing a lot of reading. I'm reading German classics... Thomas Mann and stuff like that... I enjoy it, um... I also need it for my studies. And um... yeah, when I'm finished, I want to focus on Russian literature."

It is true,
- <u>that Hannes has read more German than Russian literature so far.</u>
- that Hannes has already completed his studies and reads a lot in his free time.
- that Hannes is studying philosophy, for which he now has to read classics.

Narration 2 - Neutral & ToM

"Uh... Katharina gave me two expensive musical tickets. And I exchanged them and then told her that it was great... and then her smile froze a bit."

Hannes thinks
- <u>that Katharina wanted them to go to the musical together.</u>
- that Katharina didn't believe him that he liked the musical so much.
- that he would certainly have liked the play.

Narration 3 - Negative & noToM

"I got beaten up pretty bad on the subway... by three guys. They stood around me and kicked me. And nobody helped me."

It is true,
- <u>that Hannes couldn't run away from the thugs.</u>
- that Hannes bumped into the men on the subway and they then beat him up.
- that Hannes screamed for help when the men beat him up.

Narration 4 - Negative & ToM

"My father is an alcoholic. He often doesn't come home for days... and then the police bring him. And my mother worries like crazy. But... I'm worried too... But I can't go home every time..."

Hannes thinks
- <u>that his mother expects him to take more care of her.</u>
- that his father knows how much his mother worries about him.
- that his father will always come home at some point.

Sandra (30 years old)

Narration 1 - Neutral & noToM

“We spent two weeks at the North Sea, the weather was good, and the wind was okay. I did a lot of cycling. The water was still a bit too cold for me. But my boyfriend went in several times anyway, so I read a lot and went for walks on the beach.”

It is true,
- <u>that her boyfriend went swimming in the North Sea several times, even though the water was cold.</u>
- that she only read when her boyfriend was swimming.
- that she went to the Baltic Sea with her boyfriend for two weeks.

Narration 2 - Neutral & ToM

“Well, I couldn't be at rehearsals for quite a while and my band mates have experimented a lot during that time and I think they want to create something like a new style. Of course, with my piano, it will sound completely different now.”

Sandra thinks
- <u>that the new pieces will change again when she adds her piano playing</u>.
- that the rehearsals were bad because the piano wasn't there.
- that the band's new style doesn't suit her piano playing.

Narration 3 - Negative & noToM

“I wanted to have children, but after the diagnosis it became clear pretty quickly that my uterus would have to be removed. I was 28 when they discovered the cancer during a check-up.”

It is true,
- <u>that it was too late for non-surgical treatment when the cancer was discovered.</u>
- that the cancer was discovered during her first screening.
- that she cannot have children because she had cervical cancer as a teenager.

Narration 4 - Negative & ToM

“We blew a tire, we all rushed to the side of the road, only my mother – she wanted to put up the warning triangle and ... then a car came speeding up. And I saw it for a second, but ... I was paralyzed.”

Sandra thinks
- <u>that she could have saved her mother by warning her.</u>
- that she feels responsible because she let her mother put up the warning triangle on her own.
- that it was reckless of her mother to put up the warning triangle.

S2 Supplementary Material for Main Analyses

S2.1 Full LMM Tables

Affect Rating – Exp. 1: Linear Mixed Model

a. Selection of Best-Fitting Model

Model	Random Slopes	Deviance	Comp.	X²	*p*	Singular*
M1	-	6206	-	-	-	NO
M2	Visibility	6205	1 vs. 2	1.25	.536	YES
M3	Valence	5796	1 vs. 3	409.85	<.001	NO
**M4**	**Valence, ToM Requirement**	**5766**	**3 vs. 4**	**30.05**	**<.001**	**NO**
**FINAL**	**M4:** Affect Rating ~ Val * Tom R * Vis + (1 | Narr) + (1 + Val + ToM R | Part)					

* Indicates whether a singularity warning occurred during model computation. If "YES," the simpler model was selected.

b. Full LMM Tables

	Reported Model					Minimal Model					Maximal Model				
	b	*SE*	df	*t*	*p*	*b*	*SE*	df	*t*	*p*	*b*	*SE*	df	*t*	*p*
Intercept	**4.354**	**0.137**	**72.73**	**31.86**	**<.001**	**4.354**	**0.135**	**70.77**	**32.15**	**<.001**	**4.354**	**0.137**	**72.82**	**31.85**	**<.001**
Val	**-3.279**	**0.269**	**73.58**	**-12.19**	**<.001**	**-3.279**	**0.176**	**47.66**	**-18.63**	**<.001**	**-3.279**	**0.269**	**73.59**	**-12.19**	**<.001**
ToM R	**-0.486**	**0.184**	**53.56**	**-2.64**	**.011**	**-0.486**	**0.176**	**47.66**	**-2.76**	**.008**	**-0.486**	**0.184**	**53.61**	**-2.64**	**.011**
Vis	-0.045	0.050	1665.68	-0.90	.367	-0.045	0.059	1740.33	-0.77	.442	-0.045	0.055	37.23	-0.83	.413
Val x ToM R	**0.935**	**0.354**	**47.33**	**2.64**	**.011**	**0.935**	**0.352**	**47.66**	**2.65**	**.011**	**0.935**	**0.354**	**47.34**	**2.64**	**.011**
Vis x Val	0.075	0.100	1665.68	0.75	.456	0.076	0.118	1740.33	0.64	.520	0.075	0.100	1628.85	0.75	.455
Vis x ToM R	-0.071	0.100	1665.68	-0.71	.481	-0.072	0.118	1740.33	-0.61	.543	-0.071	0.100	1628.85	-0.71	.480
Vis x Val x ToM R	-0.140	0.201	1665.68	-0.70	.485	-0.141	0.235	1740.33	-0.60	.550	-0.140	0.200	1628.85	-0.70	.484
*Val * Tom R * Vis + (1 | Narr) + …*	*… + (1 + Val + ToM R | Part)*					*… + (1 | Part)*					*… + (1 + Vis + Val + ToM R | Part)*				

Abbreviations: Comp. = Comparison; Val = Valence; ToM R = ToM Requirement; Vis = Visibility; Narr = Narration; Part = Participant; SE = Standard Error

Affect Rating – Exp. 2: Linear Mixed Model

a. Selection of Best-Fitting Model

Model	Random Slopes	Deviance	Comp.	X^2	*p*	Singular*
M1	-	6618	-	-	-	NO
M2	Visibility	6615	1 vs. 2	3.40	.182	YES
M3	Valence	6085	1 vs. 3	533.23	<.001	NO
**M4**	**Valence, ToM Requirement**	**6057**	**3 vs. 4**	**28.18**	**<.001**	**NO**
**FINAL**	**M4:** Affect Rating ~ Val * Tom R * Vis + (1 | Narr) + (1 + Val + ToM R | Part)					

* Indicates whether a singularity warning occurred during model computation. If "YES," the simpler model was selected.

b. Full LMM Tables

	Reported Model					Minimal Model					Maximal Model				
	b	*SE*	df	*t*	*p*	*b*	*SE*	df	*t*	*p*	*b*	*SE*	df	*t*	*p*
Intercept	**4.450**	**0.190**	**61.40**	**23.38**	**<.001**	**4.450**	**0.189**	**60.34**	**23.49**	**<.001**	**4.450**	**0.190**	**61.43**	**23.39**	**<.001**
Val	**-3.005**	**0.283**	**77.68**	**-10.63**	**<.001**	**-3.005**	**0.186**	**47.42**	**-16.14**	**<.001**	**-3.005**	**0.282**	**77.79**	**-10.64**	**<.001**
ToM R	-0.388	0.195	54.27	-1.99	.052	**-0.388**	**0.186**	**47.42**	**-2.08**	**.043**	-0.388	0.195	54.59	-1.99	.052
Vis	**-0.161**	**0.048**	**1755.04**	**-3.33**	**<.001**	**-0.161**	**0.058**	**1833.40**	**-2.77**	**.006**	**-0.161**	**0.057**	**60.94**	**-2.82**	**.006**
Val x ToM R	0.698	0.374	47.24	1.87	.068	0.698	0.372	47.42	1.87	.067	0.698	0.374	47.29	1.87	.068
Vis x Val	0.121	0.096	1755.04	1.25	.210	0.120	0.116	1833.40	1.04	.300	0.121	0.096	1755.16	1.26	.208
Vis x ToM R	0.019	0.096	1755.04	0.20	.842	0.020	0.116	1833.40	0.17	.865	0.019	0.096	1755.16	0.20	.841
Vis x Val x ToM R	0.135	0.193	1755.04	0.70	.483	0.135	0.232	1833.40	0.58	.561	0.135	0.192	1755.16	0.70	.481
*Val * Tom R * Vis + (1 | Narr) + …*	*… + (1 + Val + ToM R | Part)*					*… + (1 | Part)*					*… + (1 + Vis + Val + ToM R | Part)*				

Abbreviations: Comp. = Comparison; Val = Valence; ToM R = ToM Requirement; Vis = Visibility; Narr = Narration; Part = Participant; SE = Standard Error

Affect Rating – Exp. 3: Linear Mixed Model

a. Selection of Best-Fitting Model

Model	Random Slopes	Deviance	Comp.	X²	*p*	Singular*
M1	-	6945	-	-	-	NO
M2	Visibility	6945	1 vs. 2	0.09	.954	YES
**M3**	**Valence**	**6597**	**1 vs. 3**	**347.62**	**<.001**	**NO**
M4	Valence, ToM Requirement	6569	3 vs. 4	28.37	<.001	YES
**FINAL**	**M3:** Affect Rating ~ Val * Tom R * Vis + (1 | Narr) + (1 + Val | Part)					

* Indicates whether a singularity warning occurred during model computation. If "YES," the simpler model was selected.

b. Full LMM Tables

	Reported Model					Minimal Model					Maximal Model				
	b	*SE*	df	*t*	*p*	*b*	*SE*	df	*t*	*p*	*b*	*SE*	df	*t*	*p*
Intercept	**4.048**	**0.131**	**72.34**	**30.98**	**<.001**	**4.048**	**0.130**	**71.05**	**31.23**	**<.001**	**4.048**	**0.131**	**72.43**	**30.96**	**<.001**
Val	**-3.580**	**0.293**	**81.97**	**-12.21**	**<.001**	**-3.580**	**0.224**	**47.94**	**-15.95**	**<.001**	**-3.580**	**0.293**	**82.05**	**-12.21**	**<.001**
ToM R	**-0.499**	**0.225**	**47.76**	**-2.21**	**.032**	**-0.499**	**0.224**	**47.94**	**-2.22**	**.031**	**-0.499**	**0.230**	**51.42**	**-2.17**	**.035**
Vis	-0.100	0.054	1840.13	-1.86	.063	-0.100	0.061	1880.27	-1.66	.098	-0.100	0.054	1593.07	-1.87	.061
Val x ToM R	**0.949**	**0.451**	**47.76**	**2.11**	**.040**	**0.949**	**0.449**	**47.94**	**2.11**	**.040**	**0.949**	**0.451**	**47.75**	**2.11**	**.040**
Vis x Val	0.002	0.108	1840.13	0.02	.982	0.002	0.121	1880.27	0.02	.986	0.002	0.107	1839.99	0.02	.982
Vis x ToM R	-0.032	0.108	1840.13	-0.29	.770	-0.032	0.121	1880.27	-0.26	.793	-0.032	0.107	1839.99	-0.29	.768
Vis x Val x ToM R	-0.282	0.216	1840.13	-1.31	.192	-0.282	0.243	1880.27	-1.16	.245	-0.282	0.214	1839.99	-1.32	.188
*Val * Tom R * Vis + (1 | Narr) + …*	*… + (1 + Val | Part)*					*… + (1 | Part)*					*… + (1 + Vis + Val + ToM R | Part)*				

Abbreviations: Comp. = Comparison; Val = Valence; ToM R = ToM Requirement; Vis = Visibility; Narr = Narration; Part = Participant; SE = Standard Error

Affect Rating – Exp. 4: Linear Mixed Model

a. Selection of Best-Fitting Model

Model	Random Slopes	Deviance	Comp.	X²	*p*	Singular*
M1	-	6472	-	-	-	NO
M2	Visibility	6472	1 vs. 2	0.06	.968	NO
M3	Valence	6146	1 vs. 3	326.03	<.001	NO
**M4**	**Valence, ToM Requirement**	**6114**	**3 vs. 4**	**32.24**	**<.001**	**NO**
**FINAL**	**M4:** Affect Rating ~ Val * Tom R * Vis + (1 | Narr) + (1 + Val + ToM R | Part)					

* Indicates whether a singularity warning occurred during model computation. If "YES," the simpler model was selected.

b. Full LMM Tables

	Reported Model					Minimal Model					Maximal Model				
	b	*SE*	df	*t*	*p*	*b*	*SE*	df	*t*	*p*	*b*	*SE*	df	*t*	*p*
Intercept	**4.275**	**0.175**	**73.05**	**24.46**	**<.001**	**4.275**	**0.174**	**71.78**	**24.59**	**<.001**	**4.275**	**0.175**	**73.06**	**24.46**	**<.001**
Val	**-3.752**	**0.297**	**79.15**	**-12.65**	**<.001**	**-3.752**	**0.221**	**47.64**	**-16.98**	**<.001**	**-3.752**	**0.297**	**79.18**	**-12.65**	**<.001**
ToM R	**-0.590**	**0.230**	**53.47**	**-2.56**	**.013**	**-0.590**	**0.221**	**47.64**	**-2.67**	**.010**	**-0.590**	**0.230**	**53.68**	**-2.56**	**.013**
Vis	0.060	0.055	1665.03	1.09	.274	0.060	0.063	1739.32	0.96	.339	0.059	0.062	45.76	0.95	.345
Val x ToM R	**1.382**	**0.444**	**47.46**	**3.11**	**.003**	**1.382**	**0.442**	**47.64**	**3.13**	**.003**	**1.382**	**0.444**	**47.47**	**3.11**	**.003**
Vis x Val	-0.016	0.110	1665.03	-0.14	.886	-0.016	0.125	1739.32	-0.13	.898	-0.016	0.110	1664.12	-0.14	.885
Vis x ToM R	-0.128	0.110	1665.03	-1.16	.244	-0.128	0.125	1739.32	-1.02	.309	-0.128	0.110	1664.12	-1.17	.243
Vis x Val x ToM R	0.327	0.220	1665.03	1.49	.137	0.327	0.251	1739.32	1.30	.193	0.327	0.219	1664.12	1.49	.135
*Val * Tom R * Vis + (1 | Narr) + …*	*… + (1 + Val + ToM R | Part)*					*… + (1 | Part)*					*… + (1 + Vis + Val + ToM R | Part)*				

Abbreviations: Comp. = Comparison; Val = Valence; ToM R = ToM Requirement; Vis = Visibility; Narr = Narration; Part = Participant; SE = Standard Error

Question Accuracy – Exp. 1: Linear Mixed Model

a. Selection of Best-Fitting Model

Model	Random Slopes	Deviance	Comp.	X^2	*p*	Singular*
**M1**	**-**	**1689**	**-**	**-**	**-**	**NO**
M2	Visibility	1688	1 vs. 2	0.23	.893	NO
M3	Valence	1687	1 vs. 3	1.43	.489	YES
M4	ToM Requirement	1684	1 vs. 4	4.14	.126	NO
**FINAL**	**M1:** Accuracy ~ Val * Tom R * Vis + (1 | Narr) + (1 | Part)					

* Indicates whether a singularity warning occurred during model computation. If "YES," the simpler model was selected.

b. Full LMM Tables

	Reported Model					Minimal Model					Maximal Model				
	b	*SE*	df	*t*	*p*	*b*	*SE*	df	*t*	*p*	*b*	*SE*	df	*t*	*p*
Intercept	**0.797**	**0.022**	**58.54**	**35.60**	**<.001**	**0.797**	**0.022**	**58.54**	**35.60**	**<.001**	**0.797**	**0.022**	**58.70**	**35.54**	**<.001**
Val	**0.099**	**0.041**	**47.96**	**2.43**	**.019**	**0.099**	**0.041**	**47.96**	**2.43**	**.019**	**0.099**	**0.041**	**48.74**	**2.40**	**.020**
ToM R	0.018	0.041	47.96	0.43	.668	0.018	0.041	47.96	0.43	.668	0.018	0.042	50.21	0.42	.676
Vis	-0.016	0.017	1741.03	-0.91	.365	-0.016	0.017	1741.03	-0.91	.365	-0.016	0.018	55.17	-0.88	.384
Val x ToM R	-0.079	0.081	47.96	-0.97	.336	-0.079	0.081	47.96	-0.97	.336	-0.079	0.081	47.91	-0.97	.336
Vis x Val	-0.053	0.035	1741.03	-1.51	.132	-0.053	0.035	1741.03	-1.51	.132	-0.053	0.035	1703.40	-1.52	.130
Vis x ToM R	-0.022	0.035	1741.03	-0.63	.530	-0.022	0.035	1741.03	-0.63	.530	-0.022	0.035	1703.40	-0.63	.528
Vis x Val x ToM R	0.039	0.070	1741.03	0.55	.580	0.039	0.070	1741.03	0.55	.580	0.039	0.070	1703.40	0.56	.578
*Val * Tom R * Vis + (1 | Narr) + …*	*… + (1 | Part)*					*… + (1 | Part)*					*… + (1 + Vis + Val + ToM R | Part)*				

Abbreviations: Comp. = Comparison; Val = Valence; ToM R = ToM Requirement; Vis = Visibility; Narr = Narration; Part = Participant; SE = Standard Error

Question Accuracy – Exp. 2: Linear Mixed Model

a. Selection of Best-Fitting Model

Model	Random Slopes	Deviance	Comp.	X²	*p*	Singular*
**M1**	**-**	**1693**	**-**	**-**	**-**	**NO**
M2	Visibility	1688	1 vs. 2	4.59	.101	NO
M3	Valence	1692	1 vs. 3	0.63	.730	NO
M4	ToM Requirement	1692	1 vs. 4	0.99	.610	YES
**FINAL**	**M1:** Accuracy ~ Val * Tom R * Vis + (1 | Narr) + (1 + Val | Part)					

* Indicates whether a singularity warning occurred during model computation. If "YES," the simpler model was selected.

b. Full LMM Tables

	Reported Model					Minimal Model					Maximal Model				
	b	*SE*	df	*t*	*p*	*b*	*SE*	df	*t*	*p*	*b*	*SE*	df	*t*	*p*
Intercept	**0.803**	**0.023**	**54.74**	**34.24**	**<.001**	**0.803**	**0.023**	**54.74**	**34.24**	**<.001**	**0.803**	**0.023**	**54.89**	**34.30**	**<.001**
Val	**0.099**	**0.045**	**47.99**	**2.22**	**.031**	**0.099**	**0.045**	**47.99**	**2.22**	**.031**	**0.099**	**0.045**	**47.98**	**2.21**	**.032**
ToM R	0.003	0.045	47.99	0.07	.944	0.003	0.045	47.99	0.07	.944	0.003	0.045	48.29	0.07	.944
Vis	-0.012	0.017	1833.37	-0.74	.460	-0.012	0.017	1833.37	-0.74	.460	-0.012	0.021	38.55	-0.59	.562
Val x ToM R	0.019	0.089	47.99	0.21	.834	0.019	0.089	47.99	0.21	.834	0.019	0.089	47.84	0.21	.834
Vis x Val	0.043	0.033	1833.37	1.28	.202	0.043	0.033	1833.37	1.28	.202	0.043	0.033	1754.80	1.29	.198
Vis x ToM R	0.052	0.033	1833.37	1.55	.122	0.052	0.033	1833.37	1.55	.122	0.052	0.033	1754.80	1.56	.119
Vis x Val x ToM R	-0.068	0.067	1833.37	-1.02	.307	-0.068	0.067	1833.37	-1.02	.307	-0.068	0.066	1754.80	-1.03	.303
*Val * Tom R * Vis + (1 | Narr) + …*	*… + (1 | Part)*					*… + (1 | Part)*					*… + (1 + Vis + Val + ToM R | Part)*				

Abbreviations: Comp. = Comparison; Val = Valence; ToM R = ToM Requirement; Vis = Visibility; Narr = Narration; Part = Participant; SE = Standard Error

Question Accuracy – Exp. 3: Linear Mixed Model

a. Selection of Best-Fitting Model

Model	Random Slopes	Deviance	Comp.	X^2	*p*	Singular*
M1	-	2136	-	-	-	NO
M2	Visibility	2136	1 vs. 2	0.12	.942	NO
M3	Valence	2136	1 vs. 3	0.01	.997	YES
**M4**	**ToM Requirement**	**2120**	**1 vs. 4**	**15.60**	**<.001**	**NO**
**FINAL**	**M4:** Accuracy ~ Val * Tom R * Vis + (1 | Narr) + (1 + ToM R | Part)					

* Indicates whether a singularity warning occurred during model computation. If "YES," the simpler model was selected.

b. Full LMM Tables

	Reported Model					Minimal Model					Maximal Model				
	b	*SE*	df	*t*	*p*	*b*	*SE*	df	*t*	*p*	*b*	*SE*	df	*t*	*p*
Intercept	**0.764**	**0.020**	**60.36**	**38.19**	**<.001**	**0.764**	**0.020**	**59.86**	**38.30**	**<.001**	**0.764**	**0.020**	**60.17**	**38.22**	**<.001**
Val	0.034	0.032	47.81	1.04	.304	0.034	0.032	47.87	1.04	.303	0.034	0.032	47.63	1.04	.304
ToM R	0.048	0.036	54.65	1.31	.196	0.048	0.032	47.87	1.48	.145	0.048	0.036	54.67	1.31	.196
Vis	0.010	0.018	1840.55	0.58	.565	0.010	0.018	1881.21	0.57	.569	0.010	0.018	868.26	0.57	.566
Val x ToM R	0.002	0.065	47.81	0.03	.975	0.002	0.064	47.87	0.03	.975	0.002	0.064	47.77	0.03	.975
Vis x Val	-0.045	0.036	1840.55	-1.24	.215	-0.045	0.037	1881.21	-1.23	.220	-0.045	0.036	1834.61	-1.24	.215
Vis x ToM R	0.026	0.036	1840.55	0.73	.466	0.026	0.037	1881.21	0.72	.470	0.026	0.036	1834.61	0.73	.466
Vis x Val x ToM R	-0.100	0.073	1840.55	-1.38	.168	-0.100	0.073	1881.21	-1.37	.172	-0.100	0.073	1834.61	-1.38	.168
*Val * Tom R * Vis + (1 | Narr) + …*	*… + (1 + ToM R | Part)*					*… + (1 | Part)*					*… + (1 + Vis + Val + ToM R | Part)*				

Abbreviations: Comp. = Comparison; Val = Valence; ToM R = ToM Requirement; Vis = Visibility; Narr = Narration; Part = Participant; SE = Standard Error

Question Accuracy – Exp. 4: Linear Mixed Model

a. Selection of Best-Fitting Model

Model	Random Slopes	Deviance	Comp.	X²	*p*	Singular*
M1	-	1772	-	-	-	NO
M2	Visibility	1767	1 vs. 2	4.75	.093	NO
M3	Valence	1765	1 vs. 3	6.66	.036	YES
**M4**	**ToM Requirement**	**1764**	**1 vs. 4**	**7.64**	**.022**	**NO**
**FINAL**	**M4:** Accuracy ~ Val * Tom R * Vis + (1 | Narr) + (1 + ToM R | Part)					

* Indicates whether a singularity warning occurred during model computation. If "YES," the simpler model was selected.

b. Full LMM Tables

	Reported Model					Minimal Model					Maximal Model				
	b	*SE*	df	*t*	*p*	*b*	*SE*	df	*t*	*p*	*b*	*SE*	df	*t*	*p*
Intercept	**0.780**	**0.025**	**65.10**	**30.68**	**<.001**	**0.780**	**0.025**	**64.92**	**30.72**	**<.001**	**0.780**	**0.022**	**47.84**	**35.67**	**<.001**
Val	**0.090**	**0.044**	**47.91**	**2.06**	**.045**	**0.090**	**0.044**	**47.93**	**2.06**	**.045**	**0.090**	**0.044**	**48.69**	**2.04**	**.047**
ToM R	0.018	0.046	52.61	0.38	.702	0.018	0.044	47.93	0.40	.689	0.018	0.046	52.70	0.38	.703
Vis	0.005	0.018	1702.18	0.27	.786	0.005	0.018	1739.54	0.27	.787	0.005	0.021	40.78	0.24	.813
Val x ToM R	-0.035	0.087	47.91	-0.40	.690	-0.035	0.087	47.93	-0.40	.689	-0.035	0.087	47.84	-0.40	.690
Vis x Val	-0.009	0.035	1702.18	-0.26	.794	-0.009	0.036	1739.54	-0.26	.796	-0.009	0.036	1691.66	-0.26	.798
Vis x ToM R	0.000	0.035	1702.18	0.00	1.000	0.000	0.036	1739.54	0.00	1.000	0.000	0.036	1691.66	0.00	1.000
Vis x Val x ToM R	**0.143**	**0.071**	**1702.18**	**2.02**	**.044**	**0.143**	**0.071**	**1739.54**	**2.01**	**.045**	**0.143**	**0.072**	**1691.66**	**1.98**	**.047**
*Val * Tom R * Vis + (1 | Narr) + …*	*… + (1 + ToM R | Part)*					*… + (1 | Part)*					*… + (1 + Vis + Val + ToM R | Part)*				

Abbreviations: Comp. = Comparison; Val = Valence; ToM R = ToM Requirement; Vis = Visibility; Narr = Narration; Part = Participant; SE = Standard Error

Question RT – Exp. 1: Linear Mixed Model

a. Selection of Best-Fitting Model

Model	Random Slopes	Deviance	Comp.	X^2	*p*	Singular*
**M1**	**-**	**8880**	**-**	**-**	**-**	**NO**
M2	Visibility	8880	1 vs. 2	0.36	.835	YES
M3	Valence	8879	1 vs. 3	1.06	.590	NO
M4	ToM Requirement	8880	1 vs. 4	0.23	.889	YES
**FINAL**	**M1:** RT ~ Val * Tom R * Vis + (1 | Narr) + (1 | Part)					

* Indicates whether a singularity warning occurred during model computation. If “YES,” the simpler model was selected.

b. Full LMM Tables

	Reported Model					Minimal Model					Maximal Model				
	b	*SE*	df	*t*	*p*	*b*	*SE*	df	*t*	*p*	*b*	*SE*	df	*t*	*p*
Intercept	**17.674**	**0.642**	**60.95**	**27.53**	**<.001**	**17.674**	**0.642**	**60.95**	**27.53**	**<.001**	**17.679**	**0.644**	**60.82**	**27.45**	**<.001**
Val	0.369	0.710	46.68	0.52	.606	0.369	0.710	46.68	0.52	.606	0.363	0.721	47.80	0.50	.617
ToM R	0.098	0.710	46.70	0.14	.891	0.098	0.710	46.70	0.14	.891	0.099	0.713	46.93	0.14	.891
Vis	0.324	0.261	1361.57	1.24	.215	0.324	0.261	1361.57	1.24	.215	0.325	0.262	515.36	1.24	.216
Val x ToM R	-0.666	1.419	46.69	-0.47	.641	-0.666	1.419	46.69	-0.47	.641	-0.674	1.420	46.68	-0.47	.637
Vis x Val	0.300	0.522	1361.73	0.57	.566	0.300	0.522	1361.73	0.57	.566	0.298	0.520	1332.92	0.57	.566
Vis x ToM R	-0.342	0.521	1361.31	-0.66	.511	-0.342	0.521	1361.31	-0.66	.511	-0.333	0.519	1331.28	-0.64	.521
Vis x Val x ToM R	0.713	1.044	1361.94	0.68	.494	0.713	1.044	1361.94	0.68	.494	0.720	1.040	1330.57	0.69	.489
*Val * Tom R * Vis + (1 | Narr) + …*	*… + (1 | Part)*					*… + (1 | Part)*					*… + (1 + Vis + Val + ToM R | Part)*				

Abbreviations: Comp. = Comparison; Val = Valence; ToM R = ToM Requirement; Vis = Visibility; Narr = Narration; Part = Participant; SE = Standard Error

Question RT – Exp. 2: Linear Mixed Model

a. Selection of Best-Fitting Model

Model	Random Slopes	Deviance	Comp.	X²	*p*	Singular*
**M1**	**-**	**8729**	**-**	**-**	**-**	**NO**
M2	Visibility	8728	1 vs. 2	0.97	.617	NO
M3	Valence	8729	1 vs. 3	0.01	.996	YES
M4	ToM Requirement	8727	1 vs. 4	1.50	.473	YES
**FINAL**	**M1:** RT ~ Val * Tom R * Vis + (1 | Narr) + (1 | Part)					

* Indicates whether a singularity warning occurred during model computation. If "YES," the simpler model was selected.

b. Full LMM Tables

	Reported Model					Minimal Model					Maximal Model				
	b	*SE*	df	*t*	*p*	*b*	*SE*	df	*t*	*p*	*b*	*SE*	df	*t*	*p*
Intercept	**16.392**	**0.599**	**58.98**	**27.37**	**<.001**	**16.392**	**0.599**	**58.98**	**27.37**	**<.001**	**16.395**	**0.600**	**59.01**	**27.34**	**<.001**
Val	-0.597	0.592	45.04	-1.01	.319	-0.597	0.592	45.04	-1.01	.319	-0.602	0.599	46.47	-1.00	.320
ToM R	0.346	0.592	45.02	0.58	.562	0.346	0.592	45.02	0.58	.562	0.343	0.600	46.54	0.57	.570
Vis	0.107	0.211	1424.61	0.51	.610	0.107	0.211	1424.61	0.51	.610	0.111	0.238	49.74	0.46	.645
Val x ToM R	-1.829	1.185	45.02	-1.54	.130	-1.829	1.185	45.02	-1.54	.130	-1.843	1.186	44.99	-1.55	.127
Vis x Val	0.226	0.420	1424.08	0.54	.591	0.226	0.420	1424.08	0.54	.591	0.244	0.417	1393.01	0.59	.558
Vis x ToM R	**0.905**	**0.420**	**1423.77**	**2.15**	**.031**	**0.905**	**0.420**	**1423.77**	**2.15**	**.031**	**0.902**	**0.417**	**1394.06**	**2.16**	**.031**
Vis x Val x ToM R	-1.436	0.841	1424.14	-1.71	.088	-1.436	0.841	1424.14	-1.71	.088	-1.462	0.834	1390.30	-1.75	.080
*Val * Tom R * Vis + (1 | Narr) + …*	*… + (1 | Part)*					*… + (1 | Part)*					*… + (1 + Vis + Val + ToM R | Part)*				

Abbreviations: Comp. = Comparison; Val = Valence; ToM R = ToM Requirement; Vis = Visibility; Narr = Narration; Part = Participant; SE = Standard Error

Question RT – Exp. 3: Linear Mixed Model

a. Selection of Best-Fitting Model

Model	Random Slopes	Deviance	Comp.	X^2	*p*	Singular*
**M1**	**-**	**9074**	**-**	**-**	**-**	**NO**
M2	Visibility	9072	1 vs. 2	1.37	.505	YES
M3	Valence	9074	1 vs. 3	0.00	.999	YES
M4	ToM Requirement	9071	1 vs. 4	2.78	.249	NO
**FINAL**	**M1:** RT ~ Val * Tom R * Vis + (1 | Narr) + (1 | Part)					

* Indicates whether a singularity warning occurred during model computation. If "YES," the simpler model was selected.

b. Full LMM Tables

	Reported Model					Minimal Model					Maximal Model				
	b	*SE*	df	*t*	*p*	*b*	*SE*	df	*t*	*p*	*b*	*SE*	df	*t*	*p*
Intercept	**18.007**	**0.642**	**57.09**	**28.06**	**<.001**	**18.007**	**0.642**	**57.09**	**28.06**	**<.001**	**17.994**	**0.641**	**56.76**	**28.06**	**<.001**
Val	0.107	0.601	45.72	0.18	.860	0.107	0.601	45.72	0.18	.860	0.119	0.597	45.95	0.20	.843
ToM R	0.195	0.602	45.83	0.32	.747	0.195	0.602	45.83	0.32	.747	0.215	0.619	50.42	0.35	.730
Vis	0.153	0.253	1396.88	0.60	.547	0.153	0.253	1396.88	0.60	.547	0.147	0.262	141.54	0.56	.575
Val x ToM R	-2.384	1.202	45.72	-1.98	.053	-2.384	1.202	45.72	-1.98	.053	-2.380	1.190	45.66	-2.00	.052
Vis x Val	0.201	0.506	1396.56	0.40	.691	0.201	0.506	1396.56	0.40	.691	0.221	0.503	1370.07	0.44	.660
Vis x ToM R	0.205	0.506	1396.52	0.41	.686	0.205	0.506	1396.52	0.41	.686	0.215	0.503	1370.17	0.43	.669
Vis x Val x ToM R	-0.170	1.012	1396.45	-0.17	.866	-0.170	1.012	1396.45	-0.17	.866	-0.162	1.006	1372.09	-0.16	.872
*Val * Tom R * Vis + (1 | Narr) + …*	*… + (1 | Part)*					*… + (1 | Part)*					*… + (1 + Vis + Val + ToM R | Part)*				

Abbreviations: Comp. = Comparison; Val = Valence; ToM R = ToM Requirement; Vis = Visibility; Narr = Narration; Part = Participant; SE = Standard Error

Question RT – Exp. 4: Linear Mixed Model

a. Selection of Best-Fitting Model

Model	Random Slopes	Deviance	Comp.	X²	*p*	Singular*
**M1**	**-**	**8213**	**-**	**-**	**-**	**NO**
M2	Visibility	8211	1 vs. 2	1.90	.386	NO
M3	Valence	8212	1 vs. 3	1.32	.517	NO
M4	ToM Requirement	8212	1 vs. 4	1.00	.606	NO
**FINAL**	**M1:** RT ~ Val * Tom R * Vis + (1 | Narr) + (1 | Part)					

* Indicates whether a singularity warning occurred during model computation. If "YES," the simpler model was selected.

b. Full LMM Tables

	Reported Model					Minimal Model					Maximal Model				
	b	*SE*	df	*t*	*p*	*b*	*SE*	df	*t*	*p*	*b*	*SE*	df	*t*	*p*
Intercept	**16.497**	**0.548**	**61.27**	**30.09**	**<.001**	**16.497**	**0.548**	**61.27**	**30.09**	**<.001**	**16.490**	**0.547**	**61.73**	**30.15**	**<.001**
Val	-0.237	0.613	45.70	-0.39	.700	-0.237	0.613	45.70	-0.39	.700	-0.214	0.634	49.08	-0.34	.738
ToM R	0.038	0.613	45.75	0.06	.950	0.038	0.613	45.75	0.06	.950	0.046	0.622	46.49	0.07	.942
Vis	0.283	0.230	1316.04	1.23	.220	0.283	0.230	1316.04	1.23	.220	0.305	0.268	43.13	1.14	.261
Val x ToM R	-1.612	1.225	45.70	-1.32	.195	-1.612	1.225	45.70	-1.32	.195	-1.658	1.231	45.66	-1.35	.185
Vis x Val	0.801	0.461	1315.84	1.74	.082	0.801	0.461	1315.84	1.74	.082	0.774	0.455	1261.61	1.70	.090
Vis x ToM R	-0.060	0.460	1315.43	-0.13	.896	-0.060	0.460	1315.43	-0.13	.896	-0.084	0.455	1266.57	-0.18	.854
Vis x Val x ToM R	-0.630	0.922	1316.23	-0.68	.495	-0.630	0.922	1316.23	-0.68	.495	-0.610	0.911	1259.63	-0.67	.503
*Val * Tom R * Vis + (1 | Narr) + …*	*… + (1 | Part)*					*… + (1 | Part)*					*… + (1 + Vis + Val + ToM R | Part)*				

Abbreviations: Comp. = Comparison; Val = Valence; ToM R = ToM Requirement; Vis = Visibility; Narr = Narration; Part = Participant; SE = Standard Error

Prosociality Rating – Exp. 1: Linear Mixed Model

a. Selection of Best-Fitting Model

Model	Random Slopes	Deviance	Comp.	X^2	*p*	Singular*
M1	-	7206	-	-	-	NO
M2	Visibility	7204	1 vs. 2	1.53	.465	YES
**M3**	**Valence**	**6913**	**1 vs. 3**	**292.34**	**<.001**	**NO**
M4	Valence, ToM Requirement	6888	3 vs. 4	25.37	<.001	YES
**FINAL**	**M3:** Prosocial Rating ~ Val * Tom R * Vis + (1 | Narr) + (1 + Val | Part)					

* Indicates whether a singularity warning occurred during model computation. If "YES," the simpler model was selected.

b. Full LMM Tables

	Reported Model					Minimal Model					Maximal Model				
	b	*SE*	df	*t*	*p*	*b*	*SE*	df	*t*	*p*	*b*	*SE*	df	*t*	*p*
Intercept	**4.952**	**0.273**	**56.12**	**18.11**	**<.001**	**4.952**	**0.273**	**55.53**	**18.16**	**<.001**	**4.952**	**0.273**	**56.08**	**18.12**	**<.001**
Val	**2.995**	**0.344**	**77.88**	**8.69**	**<.001**	**2.995**	**0.254**	**47.38**	**11.80**	**<.001**	**2.995**	**0.344**	**77.99**	**8.70**	**<.001**
ToM R	**0.525**	**0.255**	**47.17**	**2.06**	**.045**	**0.525**	**0.254**	**47.38**	**2.07**	**.044**	**0.525**	**0.261**	**51.47**	**2.01**	**.049**
Vis	**-0.219**	**0.068**	**1702.78**	**-3.21**	**.001**	**-0.220**	**0.076**	**1740.11**	**-2.88**	**.004**	**-0.219**	**0.071**	**185.11**	**-3.09**	**.002**
Val x ToM R	-0.410	0.510	47.17	-0.80	.425	-0.410	0.508	47.38	-0.81	.423	-0.410	0.509	47.17	-0.81	.424
Vis x Val	-0.080	0.137	1702.78	-0.58	.560	-0.079	0.153	1740.11	-0.52	.606	-0.080	0.135	1702.56	-0.59	.556
Vis x ToM R	0.046	0.137	1702.78	0.34	.734	0.046	0.153	1740.11	0.30	.762	0.046	0.135	1702.56	0.34	.731
Vis x Val x ToM R	-0.048	0.273	1702.78	-0.18	.860	-0.047	0.305	1740.11	-0.15	.877	-0.048	0.271	1702.56	-0.18	.858
*Val * Tom R * Vis + (1 | Narr) + …*	*… + (1 + Val | Part)*					*… + (1 | Part)*					*… + (1 + Vis + Val + ToM R | Part)*				

Abbreviations: Comp. = Comparison; Val = Valence; ToM R = ToM Requirement; Vis = Visibility; Narr = Narration; Part = Participant; SE = Standard Error

Prosociality Rating – Exp. 2: Linear Mixed Model

a. Selection of Best-Fitting Model

Model	Random Slopes	Deviance	Comp.	X^2	*p*	Singular*
M1	-	7262	-	-	-	NO
M2	Visibility	7261	1 vs. 2	0.11	.947	YES
**M3**	**Valence**	**6854**	**1 vs. 3**	**407.44**	**<.001**	**NO**
M4	Valence, ToM Requirement	6841	3 vs. 4	12.98	.005	YES
**FINAL**	**M3:** Prosocial Rating ~ Val * Tom R * Vis + (1 | Narr) + (1 + Val | Part)					

* Indicates whether a singularity warning occurred during model computation. If "YES," the simpler model was selected.

b. Full LMM Tables

	Reported Model					Minimal Model					Maximal Model				
	b	*SE*	df	*t*	*p*	*b*	*SE*	df	*t*	*p*	*b*	*SE*	df	*t*	*p*
Intercept	**5.676**	**0.204**	**74.32**	**27.78**	**<.001**	**5.676**	**0.203**	**73.14**	**27.93**	**<.001**	**5.676**	**0.205**	**74.44**	**27.75**	**<.001**
Val	**2.536**	**0.342**	**81.45**	**7.43**	**<.001**	**2.536**	**0.250**	**47.61**	**10.15**	**<.001**	**2.536**	**0.342**	**81.59**	**7.42**	**<.001**
ToM R	0.272	0.251	47.40	1.08	.285	0.272	0.250	47.61	1.09	.282	0.272	0.255	49.34	1.07	.291
Vis	0.087	0.060	1794.14	1.46	.143	0.088	0.069	1833.28	1.28	.201	0.087	0.066	53.33	1.31	.195
Val x ToM R	-0.035	0.502	47.40	-0.07	.944	-0.035	0.500	47.61	-0.07	.944	-0.035	0.503	47.36	-0.07	.944
Vis x Val	0.130	0.119	1794.14	1.09	.278	0.129	0.137	1833.28	0.94	.347	0.130	0.118	1753.46	1.09	.274
Vis x ToM R	-0.005	0.119	1794.14	-0.04	.967	-0.004	0.137	1833.28	-0.03	.974	-0.005	0.118	1753.46	-0.04	.967
Vis x Val x ToM R	-0.376	0.239	1794.14	-1.58	.115	-0.377	0.275	1833.28	-1.37	.170	-0.376	0.237	1753.46	-1.59	.112
*Val * Tom R * Vis + (1 | Narr) + …*	*… + (1 + Val | Part)*					*… + (1 | Part)*					*… + (1 + Vis + Val + ToM R | Part)*				

Abbreviations: Comp. = Comparison; Val = Valence; ToM R = ToM Requirement; Vis = Visibility; Narr = Narration; Part = Participant; SE = Standard Error

Prosociality Rating – Exp. 4: Linear Mixed Model

a. Selection of Best-Fitting Model

Model	Random Slopes	Deviance	Comp.	X²	p	Singular*
M1	-	7125	-	-	-	NO
M2	Visibility	7117	1 vs. 2	8.31	.016	NO
M3	Visibility, Valence	6965	2 vs. 3	152.17	<.001	NO
**M4**	**Visibility, Valence, ToM Requirement**	**6942**	**3 vs. 4**	**23.02**	**<.001**	**NO**
**FINAL**	**M4:** Prosocial Rating ~ Val * Tom R * Vis + (1 | Narr) + (1 + Vis + Val + ToM R | Part)					

* Indicates whether a singularity warning occurred during model computation. If "YES," the simpler model was selected.

b. Full LMM Tables

	Reported Model					Minimal Model					Maximal Model				
	b	SE	df	t	p	b	SE	df	t	p	b	SE	df	t	p
Intercept	**5.377**	**0.282**	**58.58**	**19.09**	**<.001**	**5.377**	**0.281**	**58.34**	**19.10**	**<.001**	**5.377**	**0.282**	**58.58**	**19.09**	**<.001**
Val	**3.077**	**0.327**	**72.66**	**9.42**	**<.001**	**3.077**	**0.276**	**47.42**	**11.13**	**<.001**	**3.077**	**0.327**	**72.66**	**9.42**	**<.001**
ToM R	0.537	0.283	51.13	1.90	.063	0.537	0.276	47.42	1.94	.058	0.537	0.283	51.13	1.90	.063
Vis	0.030	0.093	37.73	0.32	.748	0.030	0.074	1739.31	0.40	.687	0.030	0.093	37.73	0.32	.748
Val x ToM R	-0.333	0.552	47.22	-0.60	.549	-0.333	0.553	47.42	-0.60	.549	-0.333	0.552	47.22	-0.60	.549
Vis x Val	0.080	0.137	1628.67	0.58	.560	0.080	0.149	1739.31	0.54	.593	0.080	0.137	1628.67	0.58	.560
Vis x ToM R	-0.051	0.137	1628.67	-0.37	.710	-0.051	0.149	1739.31	-0.34	.734	-0.051	0.137	1628.67	-0.37	.710
Vis x Val x ToM R	0.135	0.274	1628.67	0.49	.623	0.134	0.297	1739.31	0.45	.651	0.135	0.274	1628.67	0.49	.623
*Val * Tom R * Vis + (1 | Narr) + …*	*… + (1 + Vis + Val + ToM R | Part)*					*… + (1 | Part)*					*… + (1 + Vis + Val + ToM R | Part)*				

Abbreviations: Comp. = Comparison; Val = Valence; ToM R = ToM Requirement; Vis = Visibility; Narr = Narration; Part = Participant; SE = Standard Error

S2.2 Estimated Marginal Means

Affect Rating (Exps. 1-4) – Estimated Marginal Means

	Experiment 1				Experiment 2				Experiment 3				Experiment 4			
	Full Visibility		Eyes Covered		Full Visibility		Mouth Covered		Eyes Covered		Mouth Covered		Full Visibility		No Visibility	
	M	*SE*	*M*	*SE*	*M*	*SE*	*M*	*SE*	*M*	*SE*	*M*	*SE*	*M*	*SE*	*M*	*SE*
Overall	4.38	0.14	4.33	0.14	4.53	0.19	4.37	0.19	4.10	0.13	4.00	0.13	4.25	0.18	4.31	0.18
Neutral	6.03	0.19	5.95	0.19	6.06	0.18	5.84	0.18	5.89	0.18	5.79	0.18	6.12	0.20	6.19	0.20
noToM	6.51	0.23	6.43	0.23	6.42	0.23	6.22	0.23	6.40	0.24	6.25	0.24	6.68	0.26	6.90	0.26
ToM	5.56	0.23	5.48	0.23	5.71	0.22	5.46	0.22	5.37	0.24	5.33	0.24	5.55	0.26	5.47	0.26
Negative	2.72	0.20	2.71	0.20	3.00	0.29	2.90	0.29	2.31	0.22	2.21	0.22	2.37	0.26	2.43	0.26
noToM	2.69	0.23	2.75	0.23	3.04	0.31	2.89	0.31	2.28	0.27	2.26	0.27	2.33	0.29	2.37	0.29
ToM	2.74	0.25	2.67	0.25	2.96	0.33	2.90	0.33	2.34	0.27	2.15	0.27	2.41	0.32	2.48	0.32

Question Accuracy (Exps. 1-4) – Estimated Marginal Means

	Experiment 1				Experiment 2				Experiment 3				Experiment 4			
	Full Visibility		Eyes Covered		Full Visibility		Mouth Covered		Eyes Covered		Mouth Covered		Full Visibility		No Visibility	
	M	*SE*	*M*	*SE*	*M*	*SE*	*M*	*SE*	*M*	*SE*	*M*	*SE*	*M*	*SE*	*M*	*SE*
Overall	80.51	2.40	78.92	2.40	80.88	2.49	79.65	2.49	75.85	2.20	76.89	2.20	77.72	2.69	78.20	2.69
Neutral	74.25	3.27	75.31	3.27	76.99	3.44	73.63	3.44	73.05	2.87	76.34	2.87	72.99	3.58	73.94	3.58
noToM	70.37	4.52	73.49	4.52	79.44	4.81	71.81	4.81	72.62	4.15	72.09	4.15	69.45	5.03	73.97	5.03
ToM	78.14	4.52	77.13	4.52	74.54	4.81	75.46	4.81	73.47	3.80	80.60	3.80	76.53	4.83	73.90	4.83
Negative	86.76	3.27	82.54	3.27	84.76	3.44	85.66	3.44	78.65	2.87	77.45	2.87	82.45	3.58	82.47	3.58
noToM	87.79	4.52	83.70	4.52	84.57	4.81	84.59	4.81	75.62	4.15	75.60	4.15	84.23	5.03	80.68	5.03
ToM	85.73	4.52	81.37	4.52	84.95	4.81	86.72	4.81	81.68	3.80	79.29	3.80	80.66	4.83	84.25	4.83

Question Response Time (Exps. 1-4) – Estimated Marginal Means

	Experiment 1				Experiment 2				Experiment 3				Experiment 4			
	Full Visibility		Eyes Covered		Full Visibility		Mouth Covered		Eyes Covered		Mouth Covered		Full Visibility		No Visibility	
	M	*SE*	*M*	*SE*	*M*	*SE*	*M*	*SE*	*M*	*SE*	*M*	*SE*	*M*	*SE*	*M*	*SE*
Overall	17.51	0.65	17.84	0.66	16.34	0.61	16.45	0.61	17.93	0.65	18.08	0.65	16.36	0.56	16.64	0.56
Neutral	17.40	0.76	17.58	0.76	16.69	0.69	16.69	0.69	17.93	0.73	17.98	0.73	16.67	0.65	16.56	0.65
noToM	17.01	0.94	17.54	0.93	16.47	0.82	15.65	0.82	17.31	0.87	17.21	0.87	16.32	0.81	16.07	0.80
ToM	17.79	0.93	17.62	0.93	16.92	0.82	17.72	0.82	18.55	0.87	18.75	0.86	17.03	0.80	17.04	0.80
Negative	17.62	0.75	18.10	0.75	15.98	0.68	16.20	0.68	17.93	0.73	18.19	0.73	16.04	0.65	16.72	0.65
noToM	17.74	0.92	18.21	0.92	16.31	0.81	16.44	0.81	18.46	0.86	18.66	0.86	16.33	0.79	17.20	0.79
ToM	17.50	0.92	17.98	0.92	15.65	0.81	15.97	0.81	17.41	0.86	17.72	0.86	15.75	0.79	16.24	0.79

Prosociality Rating (Exps. 1, 2, 4) – Estimated Marginal Means

	Experiment 1				Experiment 2				Experiment 4			
	Full Visibility		Eyes Covered		Full Visibility		Mouth Covered		Full Visibility		No Visibility	
	M	*SE*	*M*	*SE*	*M*	*SE*	*M*	*SE*	*M*	*SE*	*M*	*SE*
Overall	5.06	0.28	4.84	0.28	5.63	0.21	5.72	0.21	5.36	0.30	5.39	0.27
Neutral	3.54	0.33	3.37	0.33	4.40	0.28	4.42	0.28	3.84	0.36	3.83	0.34
noToM	3.20	0.38	2.98	0.38	4.30	0.34	4.23	0.34	3.46	0.43	3.51	0.41
ToM	3.89	0.38	3.75	0.38	4.50	0.34	4.61	0.34	4.23	0.40	4.16	0.39
Negative	6.58	0.33	6.32	0.33	6.87	0.26	7.02	0.26	6.88	0.32	6.95	0.30
noToM	6.42	0.38	6.15	0.38	6.69	0.32	6.94	0.32	6.70	0.38	6.76	0.36
ToM	6.73	0.38	6.49	0.38	7.04	0.32	7.10	0.32	7.06	0.38	7.14	0.36

S3 Originally Planned Analyses

In the main text, we report linear mixed models for each experiment, thereby differing from our original analysis plan (e.g., preregistration https://doi.org/10.17605/OSF.IO/DS4VE). We initially intended to calculate repeated-measures ANOVAs for individual experiments and then pool data across experiments for combined LMMs. However, based on constructive peer-review feedback, we reconsidered this approach for two key reasons:

First, ANOVAs are not suited for trial-level analysis and cannot adequately account for participant- and stimulus-related variability. We therefore conducted more powerful experiment-wise LMMs instead.

Second, pooling data for combined LMMs presented potential confounds due to nested data structures, as conditions across experiments may not be directly comparable. For instance, evaluating covered eyes versus covered mouths in comparison to covered eyes versus fully visible face could introduce context-dependent effects that complicate pooled analysis.

Due to these methodological considerations, we focused on experiment-wise LMMs for our main analyses. Importantly, results remained largely consistent across different analytical approaches and did not lead to substantively different conclusions. In the following, we report our pre-planned analysis strategy and results.

S3.1 Analyses of Variance

3.1.1 Methods

Each experiment compared two of the overall four visibility conditions: Exp. 1: full visibility vs. eyes covered; Exp. 2: full visibility vs. mouth covered; Exp. 3: eyes covered vs. mouth covered; Exp. 4: full visibility vs. no visibility (audio-only). Combined with the Valence (neutral vs. negative) and ToM Requirement (noToM vs. noToM) manipulation of the EmpaToM, this resulted in a 2x2x2 within-subjects design per experiment. We conducted separate analyses for each experiment, applying repeated-measures ANOVAs and follow-up *t*-tests. For the comparisons most central to our research question, we additionally computed Bayes Factors for the null hypotheses (BF_{01}) using Bayesian *t*-tests (Rouder et al., 2009) with a prior distribution value of 0.707. The analyses were performed in R, version 4.3.1(R Core Team, 2023), with the help of the packages ‘afex’ (Singmann et al., 2023), ‘rstatix’ (Kassambara, 2023), ‘emmeans’ (Lenth, 2024) and ‘BayesFactor’ (Morey & Rouder, 2024).

Kassambara, A. (2023). *rstatix: Pipe-friendly framework for basic statistical tests*. https://CRAN.R-project.org/package=rstatix
Lenth, R. V. (2024). *emmeans: Estimated marginal means, aka least-squares means*. https://CRAN.R-project.org/package=emmeans
Morey, R. D., & Rouder, J. N. (2024). *BayesFactor: Computation of Bayes Factors for common designs*. https://CRAN.R-project.org/package=BayesFactor
R Core Team. (2023). *R: A language and environment for statistical computing*. R Foundation for Statistical Computing. https://www.R-project.org/
Rouder, J. N., Speckman, P. L., Sun, D., Morey, R. D., & Iverson, G. (2009). Bayesian t tests for accepting and rejecting the null hypothesis. *Psychonomic Bulletin & Review*, *16*(2), 225–237. https://doi.org/10.3758/PBR.16.2.225
Singmann, H., Bolker, B., Westfall, J., Aust, F., & Ben-Shachar, M. S. (2023). *afex: Analysis of factorial experiments*. https://CRAN.R-project.org/package=afex

3.1.2 Results

Affect Rating

Mean affect ratings for all experimental conditions are displayed in Figure S1. Negative narrations consistently elicited lower affect ratings compared to neutral ones across all four experiments, *F*s > 188.02, *p*s < .001, $\eta^2 > .83$, confirming the effectiveness of our valence manipulation. Comparing ToM trials and factual reasoning (noToM) trials, affect ratings showed no significant differences following negative narrations (*t*s < |1.54|, *p*s > .132, *d*s < |0.25|). However, after neutral narrations, participants reported lower affect in ToM compared to noToM conditions (*t*s > 6.40, *p*s < .001, *d*s > 1.01). This pattern manifested in significant Valence x ToM Requirement interactions

and main effects of ToM Requirement, *F*s > 27.71, *p*s < .001, η² > .42, consistent with previous research findings.

Regarding our central research question, we found no significant Visibility x Valence interaction in any experiment, *F*s < 1.06, *p*s > .310, η² < .03, BF_{01} > 3.58. This suggests that participants' empathic responses toward narrators remained unaffected by whether or which facial features were visible. However, we did observe significant main effects of Visibility in Experiment 2, *F*(1, 39) = 8.23, *p* = .007, η² = .17, and Experiment 3, *F*(1, 40) = 5.00, *p* = .031, η² = .11. Specifically, participants reported overall lower affect when the narrator's mouth was covered compared to either full face visibility (Experiment 2) or covered eyes (Experiment 3). No other main effects or interactions reached significance, *F*s < 1.55, *p*s > .221, η² < .04.

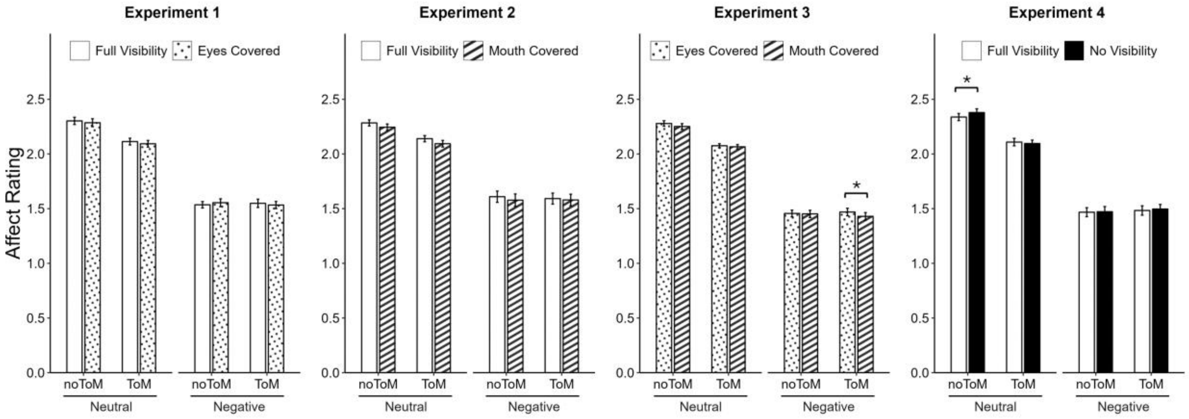

Figure S1. Mean affect ratings for Experiments 1-4. Differences between the rated affect after negative versus neutral narrations were interpreted as empathic responding. Error bars represent standard errors. On the x-axis, the different combinations of Valence and ToM Requirement are displayed. Bar colors and patterns denote Visibility condition (full visibility: white; eyes covered: dots; mouth covered: diagonal stripes; no visibility: black). Significant differences between Visibility conditions within each Valence x ToM Requirement combination are marked with horizontal brackets. *: *p* < .05.

Question Accuracy

Mean question accuracy for all conditions is presented in Figure S2. The difference between ToM and noToM questions did not reach statistical significance in any experiment, *F*s < 3.65, *p*s > .063, η² < .08. Questions following negative narrations were answered more accurately than those following

neutral narrations, as evidenced by significant main effects of Valence across all experiments *F*s > 4.53, *p*s < .039, $\eta^2 > .10$. No other main effects or interactions reached significance, *F*s < 4.07, *p*s > .051, $\eta^2 < .10$.

Crucially for our hypotheses, we found no significant main effects of Visibility, *F*s < 0.45, *p*s > .504, $\eta^2 < .01$, $BF_{01} > 4.62$, or Visibility x ToM Condition interactions, *F*s < 2.97, *p*s > .093, $\eta^2 < .07$, $BF_{01} > 1.52$, in any experiment. These results suggest that mentalizing performance was unaffected by the Visibility condition.

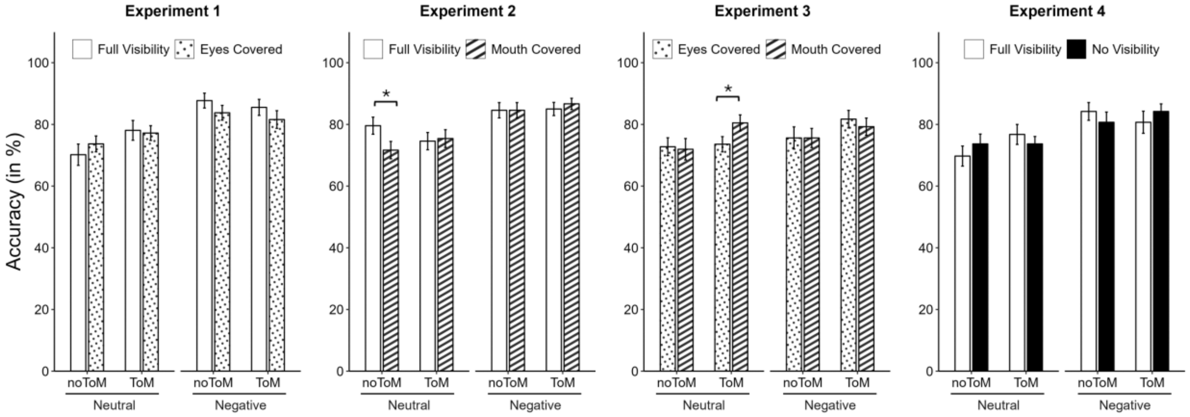

Figure S2. Mean question accuracy for Experiments 1-4. Error bars represent standard errors. On the x-axis, the different combinations of Valence and ToM Requirement are displayed. Bar colors and patterns denote Visibility condition (full visibility: white; eyes covered: dots; mouth covered: diagonal stripes; no visibility: black). Significant differences between Visibility conditions within each Valence x ToM Requirement combination are marked with horizontal brackets. *: *p* < .05.

Question Response Time

Analysis of RTs for correctly answered questions revealed significant Valence x ToM Requirement interactions in Experiments 2, 3, and 4, *F*s > 14.64, *p*s < .001, $\eta^2 > .27$. For neutral narrations, participants took longer to answer ToM questions compared to noToM questions (*t*s > |3.29|, *p*s < .002, *d*s > |0.51|). This pattern was numerically reversed in the negative condition, though only approaching statistical significance (*t*s > |1.87|, *p*s < 0.069, *d*s > |.29|). No other main effects or interactions reached significance, *F*s < 3.77, *p*s > .059, $\eta^2 < .09$.

Critically for our research questions, narrator visibility did not significantly affect RTs, as evidenced by non-significant main effects of Visibility, *F*s < 1.32, *p*s > .259, $\eta^2 < .04$, $BF_{01} > 3.69$, and non-significant ToM Condition x Visibility interactions, *F*s < 2.96, *p*s > .093, $\eta^2 < .07$, $BF_{01} > 1.52$.

Prosociality Rating

Figure S3 displays mean prosociality ratings for Experiments 1, 2, and 4. Consistent with previous findings, participants showed greater willingness to invest resources to help narrators recounting negative experiences compared to neutral ones, as indicated by significant main effects of Valence, *F*s > 111.25, *p*s < .001, η^2 > .74. Additionally, prosociality ratings were significantly higher following ToM versus noToM narrations, *F*s > 16.38, *p*s < .001, η^2 > .30. In Experiments 1 and 4, this difference was more pronounced in the neutral condition (*t*s > |5.19|, *p*s < .001, *d*s > |0.84|) than in the negative condition (*t*s > |2.93|, *p*s < .006, *d*s > |0.48|), as evidenced by significant Valence x ToM Condition interactions, *F*s > 5.51, *p*s < .024, η^2 > .13.

Regarding our central hypotheses, the main effect of Visibility reached significance in Experiment 1, *F*(1, 37) = 5.80, *p* = .021, η^2 = .14, BF_{01} = 0.45, where participants reported less willingness to help narrators with covered eyes compared to fully visible faces. In contrast, Experiments 2 and 4 showed no significant effect of Visibility on prosociality (*F*s < 2.27, *p*s > .140, η^2 < .06, BF_{01} > 2.07). No further main effects or interactions reached significance, *F*s < 2.30, *p*s > .138, η^2 < .06, including the interaction between Visibility and Valence, *F*s < 0.70, *p*s > .408, η^2 < .02, BF_{01} > 4.22.

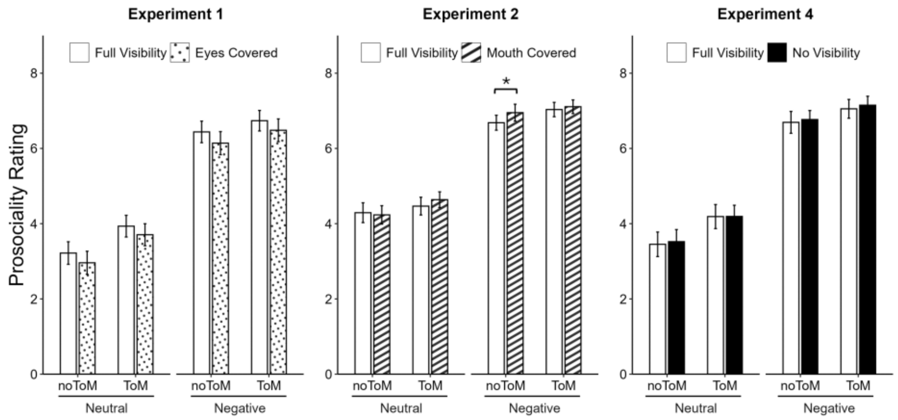

Figure S3. Mean prosociality rating for Experiments 1, 2, and 4. Error bars represent standard errors. On the x-axis, the different combinations of Valence and ToM Requirement are displayed. Bar colors and patterns denote Visibility condition (full visibility: white; eyes covered: dots; mouth covered: diagonal stripes; no visibility: black). Significant differences between Visibility conditions within each Valence x ToM Requirement combination are marked with horizontal brackets. *: *p* < .05.

S3.1.3 Supplementary Tables

Affect Rating

a. Means and Standard Errors (Exps. 1-4)

	Experiment 1				Experiment 2				Experiment 3				Experiment 4			
	Full Visibility		Eyes Covered		Full Visibility		Mouth Covered		Eyes Covered		Mouth Covered		Full Visibility		No Visibility	
	M	*SE*	*M*	*SE*	*M*	*SE*	*M*	*SE*	*M*	*SE*	*M*	*SE*	*M*	*SE*	*M*	*SE*
Overall	4.37	0.16	4.33	0.16	4.53	0.16	4.37	0.16	4.10	0.16	4.00	0.16	4.25	0.18	4.30	0.19
Neutral	6.04	0.12	5.95	0.13	6.06	0.11	5.84	0.11	5.88	0.10	5.79	0.10	6.12	0.13	6.19	0.14
noToM	6.51	0.16	6.43	0.18	6.42	0.14	6.22	0.15	6.39	0.12	6.26	0.13	6.69	0.16	6.89	0.17
ToM	5.57	0.15	5.47	0.15	5.70	0.14	5.47	0.15	5.38	0.09	5.33	0.10	5.54	0.17	5.48	0.16
Negative	2.71	0.12	2.72	0.12	3.00	0.18	2.89	0.19	2.31	0.11	2.20	0.12	2.38	0.15	2.42	0.16
noToM	2.68	0.15	2.77	0.18	3.05	0.26	2.89	0.28	2.28	0.15	2.26	0.16	2.34	0.21	2.36	0.24
ToM	2.74	0.19	2.67	0.16	2.96	0.25	2.90	0.26	2.35	0.17	2.15	0.17	2.42	0.21	2.48	0.21

b. Full ANOVA Tables (Exps. 1-4)

	Experiment 1					Experiment 2					Experiment 3					Experiment 4				
	df		*F*	*p*	η_p^2	df		*F*	*p*	η_p^2	df		*F*	*p*	η_p^2	df		*F*	*p*	η_p^2
Val	1	37	243.29	<.001	.87	1	39	188.02	<.001	.83	1	40	332.33	<.001	.89	1	37	332.65	<.001	.90
ToM R	1	37	46.09	<.001	.55	1	39	27.71	<.001	.42	1	40	52.00	<.001	.57	1	37	51.74	<.001	.58
Vis	1	37	0.55	.463	.01	1	39	8.23	.007	.17	1	40	5.00	.031	.11	1	37	0.84	.366	.02
Val x ToM R	1	37	58.37	<.001	.61	1	39	34.50	<.001	.47	1	40	73.20	<.001	.65	1	37	65.20	<.001	.64
Vis x Val	1	37	0.74	.395	.02	1	39	1.06	.310	.03	1	40	0.02	.898	.00	1	37	0.05	.819	.00
Vis x ToM R	1	37	1.00	.324	.03	1	39	0.08	.777	.00	1	40	0.13	.719	.00	1	37	0.87	.356	.02
Vis x Val x ToM R	1	37	0.56	.459	.01	1	39	0.43	.518	.01	1	40	1.45	.236	.03	1	37	1.55	.221	.04

Abbreviations: M = Mean; SE = Standard Error of the Mean; Val = Valence; ToM R = ToM Requirement; Vis = Visibility

Question Accuracy

a. Means and Standard Errors (Exps. 1-4)

	Experiment 1				Experiment 2				Experiment 3				Experiment 4			
	Full Visibility		Eyes Covered		Full Visibility		Mouth Covered		Eyes Covered		Mouth Covered		Full Visibility		No Visibility	
	M	*SE*	*M*	*SE*	*M*	*SE*	*M*	*SE*	*M*	*SE*	*M*	*SE*	*M*	*SE*	*M*	*SE*
Overall	80.37	1.56	79.06	1.31	80.94	1.31	79.58	1.35	75.91	1.50	76.83	1.51	77.85	1.67	78.07	1.46
Neutral	74.12	2.39	75.44	1.75	77.08	1.98	73.54	2.00	73.17	1.89	76.22	2.20	73.25	2.32	73.68	1.98
noToM	70.18	3.45	73.68	2.56	79.58	2.77	71.67	2.81	72.76	2.90	71.95	3.46	69.74	3.26	73.68	3.18
ToM	78.07	3.21	77.19	2.39	74.58	2.80	75.42	2.86	73.58	2.47	80.49	2.60	76.75	3.24	73.68	2.40
Negative	86.62	1.77	82.68	1.86	84.79	1.63	85.62	1.53	78.66	2.30	77.44	2.07	82.46	2.29	82.46	2.04
noToM	87.72	2.41	83.77	2.39	84.58	2.49	84.58	2.49	75.61	3.59	75.61	3.09	84.21	2.88	80.70	3.30
ToM	85.53	2.60	81.58	2.87	85.00	2.14	86.67	1.81	81.71	2.84	79.27	2.78	80.70	3.58	84.21	2.43

b. Full ANOVA Tables (Exps. 1-4)

	Experiment 1					Experiment 2					Experiment 3					Experiment 4				
	df		*F*	*p*	η_p^2	df		*F*	*p*	η_p^2	df		*F*	*p*	η_p^2	df		*F*	*p*	η_p^2
Val	1	37	30.47	<.001	.45	1	39	31.95	<.001	.45	1	40	4.53	.039	.10	1	37	29.09	<.001	.44
ToM R	1	37	0.82	.372	.02	1	39	0.04	.852	.00	1	40	3.65	.063	.08	1	37	0.62	.434	.02
Vis	1	37	0.45	.504	.01	1	39	0.35	.557	.01	1	40	0.20	.660	.00	1	37	0.01	.913	.00
Val x ToM R	1	37	4.07	.051	.10	1	39	0.31	.581	.01	1	40	0.00	.949	.00	1	37	0.91	.347	.02
Vis x Val	1	37	2.12	.154	.05	1	39	2.01	.164	.05	1	40	1.01	.321	.02	1	37	0.01	.915	.00
Vis x ToM R	1	37	0.49	.487	.01	1	39	2.97	.093	.07	1	40	0.62	.435	.02	1	37	0.00	1.000	.00
Vis x Val x ToM R	1	37	0.34	.563	.01	1	39	1.32	.258	.03	1	40	1.67	.204	.04	1	37	2.47	.124	.06

Abbreviations: M = Mean; SE = Standard Error of the Mean; Val = Valence; ToM R = ToM Requirement; Vis = Visibility

Question Response Time

a. Means and Standard Errors (Exps. 1-4)

	Experiment 1				Experiment 2				Experiment 3				Experiment 4			
	Full Visibility		Eyes Covered		Full Visibility		Mouth Covered		Eyes Covered		Mouth Covered		Full Visibility		No Visibility	
	M	*SE*	*M*	*SE*	*M*	*SE*	*M*	*SE*	*M*	*SE*	*M*	*SE*	*M*	*SE*	*M*	*SE*
Overall	17.34	0.33	17.62	0.34	16.05	0.30	16.19	0.32	17.79	0.36	17.85	0.34	16.11	0.29	16.37	0.29
Neutral	17.28	0.50	17.38	0.49	16.27	0.43	16.25	0.45	17.75	0.49	17.65	0.52	16.23	0.46	16.11	0.38
noToM	16.96	0.72	17.26	0.66	16.05	0.61	15.20	0.65	17.04	0.71	16.79	0.71	15.59	0.57	15.62	0.47
ToM	17.62	0.70	17.49	0.75	16.50	0.60	17.29	0.60	18.47	0.66	18.51	0.74	16.90	0.71	16.61	0.58
Negative	17.40	0.42	17.86	0.47	15.83	0.41	16.12	0.45	17.83	0.53	18.05	0.46	15.99	0.36	16.63	0.44
noToM	17.57	0.62	18.00	0.62	16.17	0.56	16.39	0.71	18.37	0.80	18.37	0.64	16.16	0.52	17.07	0.61
ToM	17.23	0.58	17.72	0.70	15.49	0.61	15.86	0.56	17.30	0.70	17.74	0.65	15.82	0.51	16.19	0.63

b. Full ANOVA Tables (Exps. 1-4)

	Experiment 1					Experiment 2					Experiment 3					Experiment 4				
	df		*F*	*p*	η_p^2	df		*F*	*p*	η_p^2	df		*F*	*p*	η_p^2	df		*F*	*p*	η_p^2
Val	1	36	0.82	.372	.02	1	39	1.44	.237	.04	1	40	0.73	.397	.02	1	36	0.09	.760	.00
ToM R	1	36	0.14	.708	.00	1	39	2.01	.164	.05	1	40	1.07	.306	.03	1	36	0.73	.398	.02
Vis	1	36	1.32	.259	.04	1	39	0.38	.539	.01	1	40	0.05	.829	.00	1	36	0.37	.545	.01
Val x ToM R	1	36	1.23	.274	.03	1	39	14.64	<.001	.27	1	40	15.68	<.001	.28	1	36	18.74	<.001	.34
Vis x Val	1	36	0.77	.386	.02	1	39	0.41	.527	.01	1	40	0.27	.605	.01	1	36	2.43	.128	.06
Vis x ToM R	1	36	0.41	.524	.01	1	39	2.96	.093	.07	1	40	0.49	.490	.01	1	36	0.94	.339	.03
Vis x Val x ToM R	1	36	0.34	.562	.01	1	39	3.77	.059	.09	1	40	0.02	.900	.00	1	36	0.11	.747	.00

Abbreviations: M = Mean; SE = Standard Error of the Mean; Val = Valence; ToM R = ToM Requirement; Vis = Visibility

Prosociality Rating

a. Means and Standard Errors (Exps. 1, 2, 4)

	Experiment 1				Experiment 2				Experiment 4			
	Full Visibility		Eyes Covered		Full Visibility		Mouth Covered		Full Visibility		No Visibility	
	M	*SE*	*M*	*SE*	*M*	*SE*	*M*	*SE*	*M*	*SE*	*M*	*SE*
Overall	5.08	0.19	4.82	0.19	5.62	0.15	5.73	0.15	5.35	0.19	5.41	0.19
Neutral	3.58	0.21	3.33	0.21	4.38	0.18	4.44	0.16	3.82	0.23	3.86	0.22
noToM	3.22	0.30	2.96	0.31	4.29	0.26	4.23	0.24	3.45	0.33	3.52	0.32
ToM	3.93	0.29	3.71	0.29	4.47	0.24	4.64	0.21	4.19	0.32	4.19	0.30
Negative	6.59	0.20	6.31	0.21	6.86	0.14	7.03	0.14	6.87	0.19	6.96	0.17
noToM	6.44	0.29	6.14	0.31	6.68	0.20	6.95	0.23	6.69	0.29	6.77	0.24
ToM	6.74	0.27	6.48	0.30	7.03	0.19	7.11	0.18	7.05	0.25	7.15	0.24

b. Full ANOVA Tables (Exps. 1, 2, 4)

	Experiment 1					Experiment 2					Experiment 4				
	df		*F*	*p*	η_p^2	df		*F*	*p*	η_p^2	df		*F*	*p*	η_p^2
Val	1	37	151.84	<.001	.80	1	39	111.25	<.001	.74	1	37	266.89	<.001	.88
ToM R	1	37	36.99	<.001	.50	1	39	16.38	<.001	.30	1	37	34.04	<.001	.48
Vis	1	37	5.80	.021	.14	1	39	2.27	.140	.06	1	37	0.25	.620	.01
Val x ToM R	1	37	5.69	.022	.13	1	39	0.10	.756	.00	1	37	5.51	.024	.13
Vis x Val	1	37	0.04	.852	.00	1	39	0.70	.408	.02	1	37	0.11	.741	.00
Vis x ToM R	1	37	0.07	.786	.00	1	39	0.02	.884	.00	1	37	0.02	.890	.00
Vis x Val x ToM R	1	37	0.00	.962	.00	1	39	2.30	.138	.06	1	37	0.12	.731	.00

Abbreviations: M = Mean; SE = Standard Error of the Mean; Val = Valence; ToM R = ToM Requirement; Vis = Visibility

S3.2 Linear Mixed Models on Pooled Data

3.2.1 Methods

In additional analyses, we sought to maximize statistical power through the use of LMMs on the combined data from multiple experiments. Our primary interest was to evaluate whether a (partial or complete) reduction in face visibility negatively impacted social understanding compared to unencumbered circumstances with fully visible faces. We therefore pooled the data from all experiments that contrasted full visibility with different conditions of reduced face visibility (Exp.1: full visibility vs. eyes covered, Exp.2: full visibility vs. mouth covered, Exp.4: full visibility vs. no visibility) and fitted LMMs to the trial-wise data (R packages 'lme4', Bates et al., 2015, and 'lmerTest', Kuznetsova et al., 2017), employing Maximum Likelihood estimation and Satterthwaite approximation for the degrees of freedom.

In each model, we included Level 1 fixed effects for Valence (coded as neutral = -0.5, negative = 0.5) and ToM Requirement (noToM = -0.5, ToM = 0.5). Additionally, we modeled Full Visibility (fully visible vs. covered) as a dichotomous Level 1 factor across experiments (full visibility = -0.5, eyes covered/mouth covered/no visibility = 0.5). This procedure enabled the high-powered comparison that was central to our research question (does a reduction of full visibility lead to reduced social affect and cognition?), while avoiding the issue of numerous empty cells that would arise from incorporating Visibility as a four-level within-subject factor (given that each participant encountered only two levels). To still account for different types of coverage, we introduced the Level 2 factor Experiment with two orthogonal contrasts: First, 'Coverage Extent' compared experiments featuring *partial* face coverage (Exps. 1 and 2) to *complete* face coverage (Exp.4) (Exp.1 = -0.333, Exp.2 = -0.333, Exp.4 = 0.667). If the effect of Full Visibility (fully visible vs. covered) significantly varied depending on whether some parts of the face remained visible or not, this would be evident in interactions between Full Visibility and Coverage Extent. Secondly, the contrast 'Partial Coverage Area' compared experiments with partial coverage conditions (Exp.1: eyes covered vs. Exp.2: mouth covered), which differed only in the location of the covered area (Exp.1 = -0.5, Exp.2 = 0.5, Exp.4 = 0.0). Interactions involving Full Visibility (fully visible vs. covered) and Partial Coverage Area (eyes vs. mouth) would indicate that the effect of Full Visibility specifically depended on whether the black bar covered the eyes or the mouth. Overall, our models included four fixed effects – Valence, ToM Requirement, Full Visibility, and Experiment (with contrasts for Coverage Extent and Partial Coverage Area) – along with all possible interactions among these factors.

For each of the four dependent variable, we started with a basic model which included only the specified fixed effects and a random intercept for participants. We then incrementally added random slopes for Full Visibility, Valence, and ToM Requirement and conducted Likelihood Ratio Tests to evaluate changes in the model deviance. Only random slopes that significantly improved model fit without causing singularity issues were retained. In the following results section (3.3.2), we present the findings from the model with the best fit for each dependent variable. Information on the selection of our final models is available in the supplementary tables (3.3.3).

For the sake of conciseness, we report only results of the fixed effects that are relevant to our research question. This includes the main effects of Valence and ToM Requirement as well as their interaction, which pertain to general empathic responding and perspective taking. Most importantly, we will present the results for the main effect of Full Visibility and its interaction with Valence. For between-experiment comparison, we will limit ourselves to the interactions involving Full Visibility x Coverage Extent as well as Full Visibility x Partial Coverage Area (up to three-way interactions) and will not discuss overall differences between experiments that do not relate to our main focus. For each of these effects, we report unstandardized coefficients (*b*), as well as the associated *t*- and *p*- values. Complete details on all effects are available in the supplementary tables (3.3.3).

Bates, D., Mächler, M., Bolker, B., & Walker, S. (2015). Fitting Linear Mixed-Effects Models using lme4. *Journal of Statistical Software*, *67*(1), 1–48. https://doi.org/10.18637/jss.v067.i01

Kuznetsova, A., Brockhoff, P. B., & Christensen, R. H. B. (2017). lmerTest package: Tests in Linear Mixed Effects Models. *Journal of Statistical Software*, *82*(13), 1–26. https://doi.org/10.18637/jss.v082.i13

3.2.2 Results

Affect Rating

For affect ratings, the best-fitting model included random slopes for Valence and ToM Requirement, in addition to a random intercept for participants. Consistent with the experiment-wise analyses and earlier studies, affect ratings were lower following negative narrations (Valence: $b = -3.35$, $t = -27.67$, $p < .001$) and narrations requiring mentalizing (ToM Requirement: $b = -0.49$, $t = -11.29$, $p < .001$), with a significant interaction indicating that ToM Requirement reduced affect ratings in neutral ($t(309) = 17.90$, $p < .001$) but not negative ($t(309) = -0.26$, $p = .795$) trials (Valence x ToM Requirement: $b = 1.00$, $t = 14.54$, $p < .001$). The overall affect level (regardless of Valence) did not significantly differ between narrations with uncovered and covered facial features (Full Visibility:

b = -0.05, *t* = -1.44, *p* = .151), but the pattern varied depending on whether the coverage was partial or complete (Full Visibility x Coverage Extent *b* = 0.16, *t* = 2.17, *p* = .030): While participants reported numerically lower affect when the narrator's face was partially covered compared to fully visible (significant in Exp.2, when comparing to covered mouth, t(5220) = 2.78, p = .006, but not in Exp.1, when comparing to covered eyes, t(5220) = 0.70, p = .483), they expressed no difference in affect for the complete coverage condition (i.e., audio-only) compared to full visibility (Exp. 4, *t*(5220) = -0.95, *p* = .345).

Most importantly for our research question, there was no significant interaction between Full Visibility and Valence (Full Visibility x Valence: *b* = 0.06, *t* = 0.86, *p* = .389) or between any interactions including these factors. Hence, participants' empathic resonance (i.e., tendency to feel worse after negative compared to neutral narrations) was not dependent on whether or what part of the narrator's face was visible. No other interaction of interest reached significance (*p*s > .151).

	Affect		Accuracy		RT		Prosociality	
	b	p	b	p	b	p	b	p
Valence	**-3.345**	**<.001**	**0.096**	**<.001**	-0.019	0.900	**2.869**	**<.001**
ToM Req.	**-0.488**	**<.001**	0.013	0.261	0.196	0.184	**0.445**	**<.001**
Valence x ToM Req.	**1.004**	**<.001**	-0.032	0.135	**-1.445**	**<.001**	**-0.260**	**0.003**
Full Vis.	-0.050	0.151	-0.008	0.442	0.205	0.166	-0.028	0.630
Full Vis. x Valence	0.060	0.388	-0.004	0.835	0.429	0.146	0.043	0.626
Full Vis. x ToM Req.	-0.059	0.396	0.010	0.636	0.183	0.535	0.011	0.898
Full Vis. x Valence x ToM Req.	0.096	0.488	0.038	0.375	-0.441	0.456	-0.107	0.548
Full Vis. x Cov. Extent	**0.160**	**0.030**	0.016	0.492	0.073	0.818	0.135	0.284
Full Vis. x Cov. Extent x Valence	-0.129	0.382	0.000	0.999	0.424	0.502	0.007	0.969
Full Vis. x Cov. Extent x ToM Req.	-0.083	0.573	-0.015	0.739	-0.468	0.458	-0.050	0.792
Full Vis. x Partial Cov. Area	-0.121	0.151	0.000	0.988	-0.144	0.688	**0.373**	**0.010**
Full Vis. x Partial Cov. Area x Valence	0.003	0.987	0.096	0.063	0.023	0.975	0.147	0.496
Full Vis. x Partial Cov. Area x ToM Req.	0.127	0.451	0.074	0.153	1.277	0.075	-0.019	0.932

Table S1. Unstandardized coefficient estimates (*b*) and *p*-values from the LMMs for each of the four dependent variables: affect rating, question accuracy, question RT, and prosociality rating. Significant effects are highlighted in bold. The table includes only those effects relevant to the research question. For the complete tables, refer to Supplement S3.3.3. Abbreviations: ToM Req. = ToM Requirement, Vis. = Visibility, Cov. = Coverage.

Question Accuracy

For accuracy, a model including random slopes for ToM Requirement proved to be the most appropriate in terms of model fit. In the corresponding coefficients, only the main effect of Valence – reflecting more accurate answers in the negative context – reached significance (Valence: b = 0.10, t = 9.02, p < .001). Critically, the main effect of Full Visibility was not significant (Full Visibility: b = -0.01, t = -0.77, p = .442), pointing to the robustness of mentalizing and factual reasoning to reduced visibility. There were no other significant main effects or interactions relevant to our research question (ps > .063).

Question Response Time

For RTs in trials with correct responses, no additional random slopes improved the model fit, so we retained the simple random intercept model. As in the experiment-wise analyses, there was a significant interaction between Valence and ToM Requirement (Valence x ToM Requirement: b = -1.45, t = -4.90, p < .001): Participants were faster for noToM questions in the neutral condition (t(4236) = -4.26, p <.001), but faster for ToM questions in the negative condition (t(4234) = 2.61, p = .009). No other relevant main effects or interactions reached significance (ps > .075), including the main effect of Full Visibility (b = 0.20, t = 1.39, p = .166), further supporting that ToM performance was not impaired in the absence or reduction of facial visibility.

Prosociality Rating

For prosociality ratings, the model with the best fit included random slopes for Full Visibility and Valence. In line with the experiment-wise analyses and previous findings, the main effects of Valence (Valence: b = 2.87, t = 22.23, p < .001) and ToM Requirement (ToM Requirement: b = 0.44, t = 10.01, p < .001) were significant, along with their interaction (Valence x ToM Requirement*:* b = -0.26, t = -2.92, p = .003): Participants were more willing to invest resources to help the person in the video after hearing negative narrations and narrations requiring mentalizing, with the effect of ToM Requirement being more pronounced in the neutral condition (neutral: t(5220) = -9.15, p < .001; negative: t(5220) = -5.01, p < .001).

Importantly, there was no significant main effect of Full Visibility (Full Visibility: b = -0.03, t = -0.48, p = .630), and no significant interaction between Full Visibility and Valence (Full Visibility x Valence: b = 0.04, t = 0.49, p = .626), indicating that participants’ willingness to help was, overall, not diminished when facial visibility was restricted. In the experiments examining partial coverage, the

impact of Full Visibility depended on the location of the black bar (Full Visibility x Partial Coverage Area: $b = 0.37$, $t = 2.61$, $p = .010$): Covering the narrators' eyes (Exp.1: $t(116) = 2.53$, $p = .013$) negatively impacted prosociality while covering their mouth did not (Exp.2, $t(116) = -1.14$, $p = .259$). This suggests that the decrease in willingness to help was specifically due to the eye coverage, rather than factors like the mere presence of black bars or the extent of coverage. No other relevant effects reached significance ($ps > .284$).

3.2.3 Supplementary Tables

Affect Rating - LMM on Pooled Data

a. Selection of Best-Fitting Model

Model	Random Slopes	Deviance	Comp.	X^2	*p*	Singular*
M1	-	20204	-			NO
M2	Full Visibility	20204	1 vs. 2	0.59	.744	YES
M3	Valence	19309	1 vs. 3	895.56	< .001	NO
**M4**	**Valence, ToM Requirement**	**19257**	**3 vs. 4**	**51.70**	**< .001**	**NO**
**FINAL**	**M4:** Affect Rating ~ Val * Tom R * Full Vis * Exp + (1 + Val + ToM R | Part)					

* Indicates whether a singularity warning occurred during model computation. If "YES," the simpler model was selected.

b. Full LMM Table

	b	*SE*	df	*t*	*p*
Intercept	4.360	0.081	116.00	53.84	<.001
Val	-3.345	0.121	116.00	-27.67	<.001
ToM R	-0.488	0.043	116.01	-11.29	<.001
Val x ToM R	1.004	0.069	5220.03	14.54	<.001
Full Vis	-0.050	0.035	5220.03	-1.44	0.151
Full Vis x Val	0.060	0.069	5220.03	0.86	0.388
Full Vis x ToM R	-0.059	0.069	5220.03	-0.85	0.396
Full Vis x Val x ToM R	0.096	0.138	5220.03	0.69	0.488
Cov Extent	-0.127	0.173	116.00	-0.73	0.464
Cov Extent x Val	-0.610	0.258	116.00	-2.37	0.020
Cov Extent x ToM R	-0.153	0.092	116.01	-1.66	0.100
Cov Extent x Val x ToM R	0.565	0.147	5220.03	3.84	<.001
Partial Cov Area	0.096	0.198	116.00	0.49	0.627
Partial Cov Area x Val	0.274	0.295	116.00	0.93	0.354
Partial Cov Area x ToM R	0.099	0.105	116.01	0.94	0.351
Partial Cov Area x Val x ToM R	-0.237	0.168	5220.03	-1.41	0.160
Full Vis x Cov Extent	0.160	0.074	5220.03	2.17	0.030
Full Vis x Cov Extent x Val	-0.129	0.147	5220.03	-0.88	0.382
Full Vis x Cov Extent x ToM R	-0.083	0.147	5220.03	-0.56	0.573
Full Vis x Cov Extent x Val x ToM R	0.330	0.294	5220.03	1.12	0.262
Full Vis x Partial Cov Area	-0.121	0.084	5220.03	-1.44	0.151
Full Vis x Partial Cov Area x Val	0.003	0.168	5220.03	0.02	0.987
Full Vis x Partial Cov Area x ToM R	0.127	0.168	5220.03	0.75	0.451
Full Vis x Partial Cov Area x Val x ToM R	0.286	0.337	5220.03	0.85	0.397

Abbreviations: Comp. = Comparison; Val = Valence; ToM R = ToM Requirement;
Vis = Visibility; Exp = Experiment; Part = Participant; Cov = Coverage; SE = Standard Error

Accuracy - LMM on Pooled Data

a. Selection of Best-Fitting Model

Model	Random Slopes	Deviance	Comp.	X²	*p*	Singular*
M1	-	5596	-			NO
M2	Full Visibility	5593	1 vs. 2	3.52	.172	NO
M3	Valence	5593	1 vs. 3	3.71	.156	YES
**M4**	**ToM Requirement**	**5588**	**1 vs. 4**	**8.36**	**.015**	**NO**
**FINAL**	**M4:** Accuracy ~ Val * ToM R * Full Vis * Exp + (1 + ToM R | Part)					

* Indicates whether a singularity warning occurred during model computation. If "YES," the simpler model was selected.

b. Full LMM Table

	b	*SE*	df	*t*	*p*
Intercept	0.793	0.008	116.78	104.03	<.001
Val	0.096	0.011	5452.00	9.02	<.001
ToM R	0.013	0.011	303.28	1.13	.261
Val x ToM R	-0.032	0.021	5452.00	-1.49	.135
Full Vis	-0.008	0.011	5452.00	-0.77	.442
Full Vis x Val	-0.004	0.021	5452.00	-0.21	.835
Full Vis x ToM R	0.010	0.021	5452.00	0.47	.636
Full Vis x Val x ToM R	0.038	0.043	5452.00	0.89	.375
Cov Extent	-0.020	0.016	116.78	-1.25	.215
Cov Extent x Val	-0.009	0.023	5452.00	-0.39	.694
Cov Extent x ToM R	0.007	0.024	303.28	0.30	.765
Cov Extent x Val x ToM R	-0.005	0.045	5452.00	-0.11	.912
Partial Cov Area	0.005	0.019	116.78	0.29	.770
Partial Cov Area x Val	0.000	0.026	5452.00	0.01	.992
Partial Cov Area x ToM R	-0.014	0.028	303.28	-0.52	.601
Partial Cov Area x Val x ToM R	0.098	0.052	5452.00	1.88	.060
Full Vis x Cov Extent	0.016	0.023	5452.00	0.69	.492
Full Vis x Cov Extent x Val	0.000	0.045	5452.00	0.00	.999
Full Vis x Cov Extent x ToM R	-0.015	0.045	5452.00	-0.33	.739
Full Vis x Cov Extent x Val x ToM R	0.154	0.091	5452.00	1.70	.089
Full Vis x Partial Cov Area	0.000	0.026	5452.00	-0.01	.988
Full Vis x Partial Cov Area x Val	0.096	0.052	5452.00	1.86	.063
Full Vis x Partial Cov Area x ToM R	0.074	0.052	5452.00	1.43	.153
Full Vis x Partial Cov Area x Val x ToM R	-0.115	0.104	5452.00	-1.11	.269

Abbreviations: Comp. = Comparison; Val = Valence; ToM R = ToM Requirement;
Vis = Visibility; Exp = Experiment; Part = Participant; Cov = Coverage; SE = Standard Error

Question Response Time - LMM on Pooled Data

a. Selection of Best-Fitting Model

Model	Random Slopes	Deviance	Comp.	X²	*p*	Singular*
**M1**	**-**	**26371**	**-**			**NO**
M2	Full Visibility	26371	1 vs. 2	0.57	.754	YES
M3	Valence	26371	1 vs. 3	0.03	.984	YES
M4	ToM Requirement	26371	1 vs. 4	0.08	.959	YES
**FINAL**	**M1:** RT ~ Val * ToM R * Full Vis * Exp + (1 | Part)					

* Indicates whether a singularity warning occurred during model computation. If "YES," the simpler model was selected.

b. Full LMM Table

	b	*SE*	df	*t*	*p*
Intercept	16.635	0.297	115.40	55.97	<.001
Val	-0.019	0.148	4234.45	-0.13	.900
ToM R	0.196	0.148	4235.42	1.33	.184
Val x ToM R	-1.445	0.295	4234.17	-4.90	<.001
Full Vis	0.205	0.148	4235.53	1.39	.166
Full Vis x Val	0.429	0.295	4234.37	1.45	.146
Full Vis x ToM R	0.183	0.295	4233.74	0.62	.535
Full Vis x Val x ToM R	-0.441	0.591	4234.78	-0.75	.456
Cov Extent	-0.579	0.633	115.58	-0.91	.363
Cov Extent x Val	-0.013	0.316	4234.68	-0.04	.966
Cov Extent x ToM R	-0.151	0.316	4236.26	-0.48	.632
Cov Extent x Val x ToM R	-0.182	0.632	4234.05	-0.29	.773
Partial Cov Area	-1.356	0.725	115.22	-1.87	.064
Partial Cov Area x Val	-0.688	0.358	4234.21	-1.92	.055
Partial Cov Area x ToM R	0.209	0.358	4234.55	0.58	.560
Partial Cov Area x Val x ToM R	-1.118	0.716	4234.29	-1.56	.119
Full Vis x Cov Extent	0.073	0.316	4235.79	0.23	.818
Full Vis x Cov Extent x Val	0.424	0.632	4234.84	0.67	.502
Full Vis x Cov Extent x ToM R	-0.468	0.632	4233.90	-0.74	.458
Full Vis x Cov Extent x Val x ToM R	0.160	1.265	4235.43	0.13	.899
Full Vis x Partial Cov Area	-0.144	0.359	4235.27	-0.40	.688
Full Vis x Partial Cov Area x Val	0.023	0.716	4233.89	0.03	.975
Full Vis x Partial Cov Area x ToM R	1.277	0.716	4233.58	1.78	.075
Full Vis x Partial Cov Area x Val x ToM R	-2.038	1.433	4234.10	-1.42	.155

Abbreviations: Comp. = Comparison; Val = Valence; ToM R = ToM Requirement;
Vis = Visibility; Exp = Experiment; Part = Participant; Cov = Coverage; SE = Standard Error

Prosociality Rating - LMM on Pooled Data

a. Selection of Best-Fitting Model

Model	Random Slopes	Deviance	Comp.	X²	*p*	Singular*
M1	-	22702	-			NO
M2	Full Visibility	22691	1 vs. 2	11.12	.004	NO
**M3**	**Full Visibility, Valence**	**22139**	**2 vs. 3**	**551.91**	**< .001**	**NO**
M4	Full Visibility, Valence, ToM Requirement	22102	3 vs. 4	36.71	< .001	YES
**FINAL**	**M3:** Prosocial Rating ~ Val * ToM R * Full Vis * Exp + (1 + Full Vis + Val | Part)					

* Indicates whether a singularity warning occurred during model computation. If "YES," the simpler model was selected.

b. Full LMM Table

	b	*SE*	df	*t*	*p*
Intercept	5.335	0.127	116	42.04	<.001
Val	2.869	0.129	116	22.28	<.001
ToM R	0.445	0.044	5220	10.01	<.001
Val x ToM R	-0.26	0.089	5220	-2.92	0.003
Full Vis	-0.028	0.059	116	-0.48	0.630
Full Vis x Val	0.043	0.089	5220	0.49	0.626
Full Vis x ToM R	0.011	0.089	5220	0.13	0.898
Full Vis x Val x ToM R	-0.107	0.178	5220	-0.60	0.548
Cov Extent	0.063	0.270	116	0.23	0.815
Cov Extent x Val	0.311	0.274	116	1.13	0.259
Cov Extent x ToM R	0.139	0.095	5220	1.47	0.143
Cov Extent x Val x ToM R	-0.111	0.189	5220	-0.58	0.559
Partial Cov Area	0.723	0.31	116	2.34	0.021
Partial Cov Area x Val	-0.458	0.314	116	-1.46	0.148
Partial Cov Area x ToM R	-0.253	0.108	5220	-2.34	0.019
Partial Cov Area x Val x ToM R	0.375	0.217	5220	1.73	0.084
Full Vis x Cov Extent	0.135	0.125	116	1.08	0.284
Full Vis x Cov Extent x Val	0.007	0.189	5220	0.04	0.969
Full Vis x Cov Extent x ToM R	-0.05	0.189	5220	-0.26	0.792
Full Vis x Cov Extent x Val x ToM R	0.292	0.379	5220	0.77	0.441
Full Vis x Partial Cov Area	0.373	0.143	116	2.61	0.010
Full Vis x Partial Cov Area x Val	0.147	0.217	5220	0.68	0.496
Full Vis x Partial Cov Area x ToM R	-0.019	0.217	5220	-0.09	0.932
Full Vis x Partial Cov Area x Val x ToM R	-0.434	0.433	5220	-1.00	0.317

Abbreviations: Comp. = Comparison; Val = Valence; ToM R = ToM Requirement;
Vis = Visibility; Exp = Experiment; Part = Participant; Cov = Coverage; SE = Standard Error

S4 Exploratory Analyses of the Influence of Previous Trials

Following a suggestion during the revision process, we conducted additional analyses to explore whether the condition of the previous trial influenced responses on the current trial. We would like to preface these results by noting that trial order for the different factors was randomized and not systematically controlled. As a result, the same condition (e.g., negative narrations) may have occurred in succession across several trials. Moreover, when comparing specific trial sequences (e.g., negative vs. neutral trials following a negative trial), the number of qualifying trials per participant was often small, limiting the reliability of these estimates.

We considered potential carry-over effects particularly relevant for the factors Valence and Visibility. To investigate this, we created additional variables reflecting the condition of the previous trial – Valence-Lag and Visibility-Lag, respectively – and added these to our final LMMs for the dependent variables affect rating, accuracy in the single-choice questions, and prosociality rating. For each outcome, we examined only the effects relevant to our hypotheses and their interactions with the lag variables. Specifically, for affect ratings, we probed whether the new variables interacted with the interaction between Valence and Visibility. For accuracy, we tested whether any of the new variables shaped either the main effect of ToM Requirement or the interaction between ToM Requirement and Visibility. Finally, for prosociality ratings, we focused on the new variables' interaction with Visibility as well as Visibility x Valence.

Importantly, across all models, our main effects remained stable, and no new effects emerged that would contradict our overall pattern of results or conclusions. In all but one case, there were no significant interactions between the lag variables (Valence-Lag or Visibility-Lag) and the key effects of interest. The sole exception was a significant three-way interaction between Visibility, Valence, and Visibility-Lag in the prosociality analysis of Experiment 2. This effect suggested that participants showed slightly greater willingness to help during neutral, full-visibility trials when those were preceded by a mouth-covered trial. Aside from this isolated finding, we found no indication that carry-over effects meaningfully influenced the results.

Full tables detailing the interactions between the lag variables and the effects of interest can be found in the following.

Affect Rating (Exps. 1-4): Linear Mixed Models with Lag Variables

Final models from the main analysis, extended to include fixed effects for Visibility-Lag and Valence-Lag (i.e., the condition of the previous trial)

	Experiment 1					Experiment 2				
	b	*SE*	df	*t*	*p*	*b*	*SE*	df	*t*	*p*
Val Lag	0.020	0.051	1713.43	0.38	.701	0.025	0.049	1798.52	0.50	.614
Vis Lag	0.003	0.051	1707.12	0.06	.955	0.013	0.049	1799.93	0.27	.790
Val	-3.276	0.271	73.81	-12.08	<.001	-3.009	0.283	77.20	-10.63	<.001
Val x Val Lag	-0.163	0.103	1713.42	-1.58	.113	-0.207	0.098	1786.52	-2.11	.035
Val x Vis Lag	-0.170	0.102	1707.08	-1.67	.095	-0.047	0.099	1793.92	-0.47	.637
ToM R	-0.487	0.186	53.10	-2.62	.011	-0.389	0.194	54.30	-2.01	.050
ToM R x Val Lag	0.030	0.104	1686.63	0.29	.772	0.016	0.099	1771.24	0.16	.870
ToM R x Vis Lag	0.011	0.103	1679.64	0.11	.911	-0.118	0.099	1773.51	-1.19	.233
Vis	-0.041	0.050	1663.97	-0.82	.413	-0.158	0.048	1754.30	-3.28	.001
Vis x Val Lag	-0.028	0.103	1712.03	-0.27	.790	-0.008	0.100	1805.82	-0.08	.935
Vis x Vis Lag	0.032	0.104	1716.79	0.31	.756	-0.144	0.100	1800.89	-1.44	.149
**Vis x Val**	**0.070**	**0.100**	**1664.09**	**0.70**	**.484**	**0.107**	**0.096**	**1754.12**	**1.11**	**.265**
**Vis x Val x Val Lag**	**0.049**	**0.207**	**1706.80**	**0.24**	**.812**	**-0.023**	**0.200**	**1798.03**	**-0.12**	**.907**
**Vis x Val x Vis Lag**	**-0.218**	**0.207**	**1716.44**	**-1.05**	**.293**	**-0.323**	**0.200**	**1807.13**	**-1.61**	**.107**
Vis x ToM R	-0.081	0.100	1664.15	-0.81	.417	0.022	0.096	1754.27	0.23	.822
Vis x ToM R x Val Lag	-0.533	0.206	1711.83	-2.60	.010	0.321	0.200	1801.55	1.61	.108
Vis x ToM R x Vis Lag	-0.266	0.208	1716.62	-1.28	.202	-0.119	0.200	1809.45	-0.60	.551

Note: Val = Valence; ToM R = ToM Requirement; Vis = Visibility; SE = Standard Error. Effects relevant to the hypotheses are highlighted in bold.

	Experiment 3					Experiment 4				
	b	*SE*	df	*t*	*p*	*b*	*SE*	df	*t*	*p*
Val Lag	0.045	0.055	1851.24	0.82	.410	0.012	0.057	1694.62	0.22	.828
Vis Lag	-0.025	0.055	1853.22	-0.46	.645	-0.040	0.057	1710.45	-0.71	.477
Val	-3.580	0.293	81.93	-12.23	<.001	-3.747	0.296	79.04	-12.65	<.001
Val x Val Lag	-0.171	0.110	1857.74	-1.56	.119	-0.042	0.113	1700.67	-0.37	.713
Val x Vis Lag	0.014	0.110	1859.21	0.13	.896	-0.018	0.114	1712.01	-0.16	.874
ToM R	-0.496	0.225	47.77	-2.20	.033	-0.584	0.230	53.55	-2.54	.014
ToM R x Val Lag	0.024	0.111	1867.21	0.21	.830	-0.012	0.113	1677.65	-0.10	.918
ToM R x Vis Lag	-0.127	0.111	1867.10	-1.14	.253	-0.096	0.114	1685.00	-0.84	.403
Vis	-0.099	0.054	1839.25	-1.82	.068	0.056	0.055	1664.23	1.01	.310
Vis x Val Lag	0.197	0.111	1870.30	1.77	.077	-0.064	0.115	1711.28	-0.56	.577
Vis x Vis Lag	0.042	0.111	1869.61	0.38	.707	0.037	0.114	1714.69	0.33	.744
**Vis x Val**	**0.019**	**0.108**	**1839.24**	**0.18**	**.858**	**-0.020**	**0.110**	**1664.07**	**-0.18**	**.854**
**Vis x Val x Val Lag**	**-0.064**	**0.222**	**1871.21**	**-0.29**	**.773**	**-0.059**	**0.230**	**1712.68**	**-0.25**	**.799**
**Vis x Val x Vis Lag**	**-0.009**	**0.223**	**1870.52**	**-0.04**	**.967**	**0.097**	**0.228**	**1715.27**	**0.43**	**.669**
Vis x ToM R	-0.031	0.108	1839.16	-0.29	.774	-0.137	0.110	1664.27	-1.24	.214
Vis x ToM R x Val Lag	-0.386	0.221	1858.28	-1.75	.081	0.322	0.228	1714.74	1.41	.158
Vis x ToM R x Vis Lag	0.066	0.223	1866.31	0.30	.766	-0.021	0.229	1711.15	-0.09	.928

Note: Val = Valence; ToM R = ToM Requirement; Vis = Visibility; SE = Standard Error. Effects relevant to the hypotheses are highlighted in bold.

Question Accuracy (Exps. 1-4): Linear Mixed Models with Lag Variables

Final models from the main analysis, extended to include fixed effects for Visibility-Lag and Valence-Lag (i.e., the condition of the previous trial)

	Experiment 1					Experiment 2				
	b	*SE*	df	*t*	*p*	*b*	*SE*	df	*t*	*p*
Val Lag	0.009	0.018	1756.90	0.53	.599	0.015	0.017	1842.97	0.87	.387
Vis Lag	-0.017	0.018	1749.77	-0.97	.331	-0.018	0.017	1847.39	-1.05	.296
Val	0.100	0.040	47.81	2.49	.016	0.097	0.045	47.94	2.16	.036
Val x Val Lag	-0.042	0.036	1785.39	-1.17	.243	-0.059	0.034	1866.75	-1.76	.078
Val x Vis Lag	0.047	0.035	1772.65	1.33	.182	-0.065	0.034	1880.25	-1.91	.057
ToM R	0.019	0.040	47.81	0.48	.635	0.005	0.045	47.94	0.11	.913
ToM R x Val Lag	-0.032	0.036	1785.18	-0.88	.376	-0.013	0.034	1876.88	-0.39	.693
ToM R x Vis Lag	-0.002	0.035	1774.02	-0.04	.965	-0.021	0.034	1875.86	-0.62	.534
**Vis**	**-0.017**	**0.018**	**1740.08**	**-0.96**	**.338**	**-0.014**	**0.017**	**1832.38**	**-0.86**	**.392**
**Vis x Val Lag**	**0.063**	**0.035**	**1778.78**	**1.78**	**.075**	**-0.056**	**0.034**	**1877.74**	**-1.65**	**.098**
**Vis x Vis Lag**	**-0.015**	**0.036**	**1787.08**	**-0.43**	**.664**	**-0.050**	**0.034**	**1881.27**	**-1.49**	**.137**
Vis x Val	-0.046	0.035	1739.99	-1.32	.186	0.040	0.033	1832.22	1.19	.234
Vis x Val x Val Lag	-0.109	0.071	1777.92	-1.54	.123	0.033	0.068	1880.08	0.49	.621
Vis x Val x Vis Lag	0.067	0.071	1776.29	0.94	.347	-0.019	0.068	1874.99	-0.29	.773
**Vis x ToM R**	**-0.012**	**0.035**	**1739.96**	**-0.34**	**.733**	**0.055**	**0.033**	**1832.42**	**1.66**	**.098**
**Vis x ToM R x Val Lag**	**0.049**	**0.071**	**1774.07**	**0.69**	**.490**	**0.056**	**0.068**	**1876.49**	**0.83**	**.405**
**Vis x ToM R x Vis Lag**	**0.039**	**0.071**	**1785.40**	**0.55**	**.583**	**0.097**	**0.068**	**1874.60**	**1.43**	**.152**

Note: Val = Valence; ToM R = ToM Requirement; Vis = Visibility; SE = Standard Error. Effects relevant to the hypotheses are highlighted in bold.

	Experiment 3					Experiment 4				
	b	*SE*	df	*t*	*p*	*b*	*SE*	df	*t*	*p*
Val Lag	-0.012	0.018	1888.77	-0.64	.523	-0.035	0.018	1734.10	-1.95	.051
Vis Lag	0.006	0.018	1894.98	0.30	.761	-0.009	0.018	1747.79	-0.48	.628
Val	0.036	0.033	47.75	1.09	.281	0.090	0.043	47.89	2.08	.043
Val x Val Lag	-0.022	0.037	1917.94	-0.61	.544	0.041	0.036	1764.19	1.14	.254
Val x Vis Lag	-0.023	0.037	1919.53	-0.62	.536	0.020	0.036	1778.90	0.56	.577
ToM R	0.050	0.037	55.28	1.36	.180	0.017	0.045	52.86	0.38	.705
ToM R x Val Lag	-0.006	0.037	1882.07	-0.17	.869	-0.005	0.036	1727.12	-0.14	.886
ToM R x Vis Lag	0.028	0.037	1888.26	0.77	.444	0.012	0.036	1736.72	0.33	.738
**Vis**	**0.012**	**0.018**	**1839.59**	**0.65**	**.517**	**0.004**	**0.018**	**1701.30**	**0.25**	**.802**
**Vis x Val Lag**	**0.014**	**0.037**	**1907.96**	**0.39**	**.698**	**0.010**	**0.036**	**1769.66**	**0.28**	**.779**
**Vis x Vis Lag**	**-0.025**	**0.037**	**1918.15**	**-0.69**	**.493**	**-0.002**	**0.036**	**1761.78**	**-0.07**	**.947**
Vis x Val	-0.049	0.036	1839.46	-1.34	.180	-0.005	0.035	1701.47	-0.13	.899
Vis x Val x Val Lag	-0.021	0.074	1914.56	-0.29	.771	-0.039	0.073	1771.77	-0.54	.588
Vis x Val x Vis Lag	0.191	0.074	1921.95	2.58	.010	-0.015	0.072	1771.33	-0.21	.832
**Vis x ToM R**	**0.026**	**0.036**	**1839.65**	**0.72**	**.471**	**-0.007**	**0.035**	**1701.42**	**-0.20**	**.844**
**Vis x ToM R x Val Lag**	**0.070**	**0.074**	**1908.87**	**0.95**	**.344**	**0.007**	**0.072**	**1768.96**	**0.10**	**.920**
**Vis x ToM R x Vis Lag**	**0.137**	**0.074**	**1918.28**	**1.86**	**.064**	**-0.044**	**0.072**	**1762.52**	**-0.62**	**.539**

Note: Val = Valence; ToM R = ToM Requirement; Vis = Visibility; SE = Standard Error. Effects relevant to the hypotheses are highlighted in bold.

Prosociality (Exps. 1, 2, 4): Linear Mixed Models with Lag Variables

Final models from the main analysis, extended to include fixed effects for Visibility-Lag and Valence-Lag (i.e., the condition of the previous trial)

	Experiment 1					Experiment 2				
	b	*SE*	df	*t*	*p*	*b*	*SE*	df	*t*	*p*
Val Lag	-0.014	0.070	1714.69	-0.20	.843	0.109	0.060	1800.48	1.80	.072
Vis Lag	-0.023	0.069	1710.04	-0.34	.736	0.001	0.061	1804.14	0.02	.983
Val	2.995	0.345	77.82	8.69	<.001	2.546	0.343	81.57	7.42	<.001
Val x Val Lag	-0.032	0.140	1710.07	-0.23	.820	-0.171	0.121	1799.03	-1.41	.158
Val x Vis Lag	0.253	0.139	1706.54	1.82	.068	-0.069	0.122	1801.78	-0.56	.573
ToM R	0.537	0.255	47.16	2.11	.040	0.266	0.253	47.43	1.05	.298
ToM R x Val Lag	-0.054	0.142	1717.84	-0.38	.701	-0.170	0.122	1803.67	-1.39	.166
ToM R x Vis Lag	-0.168	0.140	1712.15	-1.20	.231	0.038	0.123	1805.36	0.31	.756
**Vis**	**-0.228**	**0.068**	**1701.76**	**-3.33**	**<.001**	**0.095**	**0.059**	**1793.13**	**1.59**	**.112**
**Vis x Val Lag**	**0.047**	**0.141**	**1713.56**	**0.33**	**.739**	**0.077**	**0.123**	**1807.34**	**0.63**	**.531**
**Vis x Vis Lag**	**-0.124**	**0.141**	**1714.16**	**-0.88**	**.381**	**-0.127**	**0.123**	**1806.37**	**-1.03**	**.305**
**Vis x Val**	**-0.089**	**0.137**	**1701.77**	**-0.65**	**.513**	**0.130**	**0.119**	**1793.14**	**1.10**	**.274**
**Vis x Val x Val Lag**	**0.017**	**0.281**	**1713.73**	**0.06**	**.952**	**0.241**	**0.247**	**1807.01**	**0.98**	**.329**
**Vis x Val x Vis Lag**	**0.397**	**0.282**	**1716.49**	**1.41**	**.159**	**0.545**	**0.246**	**1807.21**	**2.21**	**.027**
Vis x ToM R	0.038	0.137	1701.76	0.27	.783	0.000	0.119	1793.13	0.00	.998
Vis x ToM R x Val Lag	-0.242	0.280	1710.86	-0.87	.386	-0.158	0.245	1804.56	-0.64	.519
Vis x ToM R x Vis Lag	-0.209	0.283	1715.55	-0.74	.460	0.157	0.246	1805.79	0.64	.522

Note: Val = Valence; ToM R = ToM Requirement; Vis = Visibility; SE = Standard Error. Effects relevant to the hypotheses are highlighted in bold.

	Experiment 4				
	b	*SE*	df	*t*	*p*
Val Lag	0.064	0.071	1713.25	0.91	.363
Vis Lag	-0.089	0.071	1707.98	-1.26	.209
Val	3.068	0.326	72.88	9.40	<.001
Val x Val Lag	-0.228	0.142	1707.25	-1.61	.107
Val x Vis Lag	-0.090	0.142	1708.84	-0.63	.526
ToM R	0.531	0.282	51.16	1.89	.065
ToM R x Val Lag	0.146	0.141	1700.29	1.03	.301
ToM R x Vis Lag	0.142	0.143	1712.80	1.00	.319
**Vis**	**0.029**	**0.092**	**37.76**	**0.31**	**.758**
**Vis x Val Lag**	**0.070**	**0.143**	**1688.08**	**0.49**	**.622**
**Vis x Vis Lag**	**-0.238**	**0.141**	**1681.85**	**-1.69**	**.092**
**Vis x Val**	**0.064**	**0.137**	**1664.98**	**0.46**	**.644**
**Vis x Val x Val Lag**	**-0.002**	**0.287**	**1716.87**	**-0.01**	**.993**
**Vis x Val x Vis Lag**	**-0.108**	**0.285**	**1704.21**	**-0.38**	**.706**
Vis x ToM R	-0.043	0.137	1664.80	-0.31	.754
Vis x ToM R x Val Lag	-0.089	0.284	1697.58	-0.31	.755
Vis x ToM R x Vis Lag	0.367	0.285	1712.05	1.29	.198

Note: Val = Valence; ToM R = ToM Requirement; Vis = Visibility; SE = Standard Error. Effects relevant to the hypotheses are highlighted in bold.
